# A *Drosophila* model for Meniere’s disease: Dystrobrevin is required for support cell function in hearing and proprioception

**DOI:** 10.3389/fcell.2022.1015651

**Published:** 2022-11-10

**Authors:** T. Requena, A. Keder, P. zur Lage, J. T. Albert, A. P. Jarman

**Affiliations:** ^1^ Biomedical Sciences: Centre for Discovery Brain Sciences, Edinburgh Medical School, University of Edinburgh, Edinburgh, United Kingdom; ^2^ Division of Functional Genetics and Development, The Royal Dick School of Veterinary Sciences, The Roslin Institute, The University of Edinburgh, Edinburgh, United Kingdom; ^3^ Ear Institute, University College London, London, United Kingdom

**Keywords:** Meniere’s disease, *Drosophila*, dystrobrevin, animal model, dystrophin, hearing

## Abstract

Meniere’s disease (MD) is an inner ear disorder characterised by recurrent vertigo attacks associated with sensorineural hearing loss and tinnitus. Evidence from epidemiology and Whole Exome Sequencing (WES) suggests a genetic susceptibility involving multiple genes, including α-Dystrobrevin (*DTNA*). Here we investigate a *Drosophila* model. We show that mutation, or knockdown, of the *DTNA* orthologue in *Drosophila*, *Dystrobrevin* (*Dyb*), results in defective proprioception and impaired function of Johnston’s Organ (JO), the fly’s equivalent of the inner ear. *Dyb* and another component of the dystrophin-glycoprotein complex (DGC), *Dystrophin* (*Dys*), are expressed in support cells within JO. Their specific locations suggest that they form part of support cell contacts, thereby helping to maintain the integrity of the hemolymph-neuron diffusion barrier, which is equivalent to a blood-brain barrier. These results have important implications for the human condition, and notably, we note that *DTNA* is expressed in equivalent cells of the mammalian inner ear.

## Introduction

Hearing loss (HL) affects about 5% of the population worldwide (World Health Organization, 2020). The aetiology of HL and inner ear disorders is diverse. It includes infections (Cohen et al., 2014), ototoxic drugs (Laurell, 2019), noise trauma (Le et al., 2017), ageing (Davis et al., 2016) and genetic factors (Korver et al., 2017). The wide-spread, and multi-factorial nature of hearing disorders exposes complex vulnerabilities on the level of the specialised mechanosensory processes, which support the ear’s function throughout life, including extensive mechanisms of auditory homeostasis. Indeed, noise-induced hearing loss (NIHL) and age-related hearing loss (ARHL) may result from acute and chronic cellular stress (respectively) that eventually overwhelm the homeostatic repair (Ohlemiller, 2006). Much of this remains uncharacterised. Meniere’s disease (MD (MIM 156000)) is a chronic disease, with an incidence of about 1 in 2000 in Caucasians (Morrison et al., 2009), characterised by episodes of vertigo, low-to-middle-frequency sensorineural hearing loss (SNHL), tinnitus and aural fullness (Lopez-Escamez et al., 2015; Lopez-Escamez et al., 2017). Most cases are sporadic, late-onset (3rd-4th decade of life) and present monaurally. A current working hypothesis is that MD results from a complex interplay of genetic susceptibility and environmental insults. In a sense, MD may be an accelerated failure in homeostatic mechanisms maintaining hearing and balance. Due to the unclear aetiology, diagnosis is complex, and there is no effective treatment. According to the clinical presentation and progression of the symptoms, there is great variability in the phenotype of MD. Correspondingly, a variety of causes and mechanisms have been theorised to underlie the specific MD pathology.

While most MD cases are sporadic, several lines of evidence suggest a genetic component in at least a subset of MD sufferers: 1) the apparent susceptibility to the disease for Caucasians over other ethnicities (Morrison et al., 2009); 2) families with MD (familial MD, fMD) represent 8%–10% of all cases in European populations (Requena et al., 2014); finally, 3) there is a high sibling recurrence risk ratio (Gazquez et al., 2012). Using a Whole Exome Sequencing (WES) approach, several genes have been linked with MD (Requena et al., 2015; Martin-Sierra et al., 2016; Martin-Sierra et al., 2017; Roman-Naranjo et al., 2020; Amanat et al., 2021), but so far, only genetic variants in the genes *DTNA*, *FAM136A* and *DPT* have been replicated in other populations (Oh et al., 2019).

**FIGURE 1:**
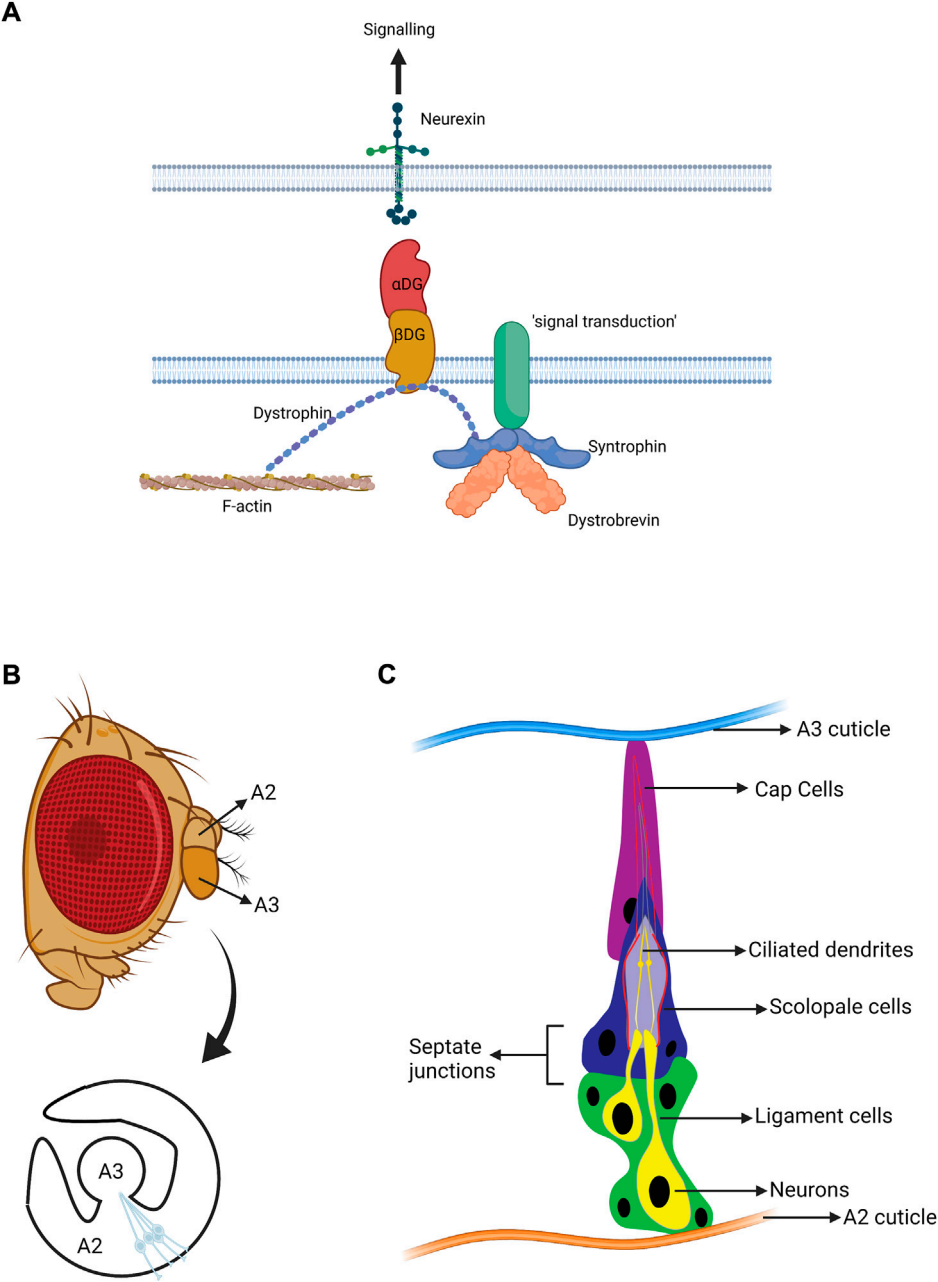
Structure of the *Drosophila* auditory organ**. (A)** Schematic diagram of the DGC. The major roles of the DGC are structural and organisation of signalling. Dystobrevin specifically participates in recruiting various signal transduction molecules. **(B)** A lateral view of an adult *Drosophila* head with a magnified view of antennal segments A2 and A3. The fly’s ear or auditory organ (Johnston’s organ, JO) is located in A2 and consists of functional units called scolopidia. These are attached to the cuticle of A2 on one side, and the A2/A3 joint on the other. The scolopidial area is highlighted, and one scolopidium is magnified. **(C)** Cellular components of an individual scolopidium. Each scolopidium is attached to the antenna cuticle by a cap cell (magenta) and ligament cells (green). The ligament cells ensheath two to three mechanosensory neurons (yellow). The ciliary dendrites of the neurons are enclosed by an actin-rich structure generated by the scolopale cell (dark blue). Scolopale cells form septate junctions with the ligament and cap cells at the basal and apical ends of the scolopidium, respectively, ensuring the formation of an enclosed space to maintain the K^+^ -rich lymph bathing the cilia. Approximately 225 scolopidia are located in JO. (B, C adapted from Li T. et al., 2018. Figures made in BioRender.com).

Recent inner ear expression data points to *DTNA* as a promising candidate for understanding the basis of MD (Orvis et al., 2021). *DTNA* encodes α-Dystrobrevin, a structural component of the dystrophin-glycoprotein complex (DGC) with isoforms predominant at the sarcolemma, synapse and neuromuscular junction (NMJ) (Pilgram et al., 2010) (Figure 1A). Suggested roles for α-Dystrobrevin within the DGC are recruitment and organisation (with syntrophins) of signalling molecules in DGC-associated cell junctions (NMJs, synapses) (Albrecht and Froehner, 2002). The wider DGC also has mechanical roles (linking ECM to the actin cytoskeleton) and cell adhesion roles, often as a partner of neurexin cell adhesion molecules. In a mouse model, the absence of α-Dystrobrevin resulted in abnormal brain capillary permeability, progressively escalating brain oedema (Lien et al., 2012). Moreover, it is expressed in the vestibular system at the early stages of development in mice, suggesting a role in the maturation of the vestibular system (Lien et al., 2004). Recent RNAseq data identifies *DTNA* expression in several inner ear cell types, including Inner and Outer Hair cells, Pillar cells, Deiter cells, and marginal and intermediate cells of the stria vascularis (Orvis et al., 2021). *DTNA’*s role in inner ear function remains undetermined, and the pathogenicity of *DTNA* mutations as a cause of MD remains unconfirmed. Moreover, the potential role of the DGC as a whole in inner ear function is unclear.

The use of *Drosophila* to model human disease is well documented (Ugur et al., 2016). The *Drosophila* hearing/balance system shares key physiological features with the mammalian inner ear, much of which reflects a common evolutionary origin, most compellingly illustrated by the multitude of shared proneural genes, such as e.g., the *atonal* homologues (ATHs) (Weinberger et al., 2017; Albert et al., 2020). Of all the genes expressed in the *Drosophila* hearing system, ∼20% have human counterparts associated with hearing disorders (Senthilan et al., 2012). Fly homologues of ATOH1, TMC1/2, MYH9 and MYO7A have played a role in understanding hearing mechanisms and are directly linked with human hearing loss (Li et al., 2018). As well as displaying congenital hearing loss, *Drosophila* hearing is subject to environmental effects relevant to other hearing issues, such as ARHL (Keder et al., 2020) and NIHL (Christie et al., 2013), both of which have been used to reveal the underlying mechanisms of homeostatic maintenance in the *Drosophila* hearing system.

The specialised biophysical and physiological mechanisms of mechanotransduction in hearing and proprioception share vast similarities across species. In vertebrates, these originate from the complex molecular transduction machinery of hair cells, which interact with ascending and descending neurons as well as supporting cells of the inner ear (Fettiplace and Hackney, 2006). In *Drosophila*, the mechanosensory and supporting cells are housed in Johnston’s Organ (JO) in the antenna, which mediates the senses of hearing, gravity, and wind detection (Figure 1B). JO is located in the 2nd antennal segment (a2) and consists of ∼225 scolopidial units. Each contains several support cells associated with 2-3 mechanosensory chordotonal neurons (Figure 1C). The ciliated neurons of JO (chordotonal neurons) are equivalent to inner ear hair cells (Kavlie and Albert, 2013). Mammalian inner ear hair cells transform sound-induced vibrations, transmitted through cochlear fluids, into electro-chemical currents through specialised transducer channels within their (microvillar) stereociliary hair bundles. Likewise, sound-induced rotations of the *Drosophila* antenna activate transducer channels in the (true) cilia of JO neurons, leading to the generation of action potentials in the fly’s antennal nerve. Despite stark differences in anatomy between fly JO and human inner ear, mechanotransduction processes display strong parallels, including conforming to a “gating spring” model, exhibiting gating compliance as well as active mechanical amplification (Albert and Kozlov, 2016).

To better understand the functions of *DTNA* and DGC in MD, we investigate the roles of the *Drosophila* homologues, Dystrobrevin (*Dyb*) and Dystrophin (*Dys*), in JO function. We show that both are required in JO for full hearing and balance functions. Interestingly, cell-type specific knockdown suggests they are required primarily in supporting cells that envelop the mechanosensory neuron, and this is supported by expression analysis. Our analysis suggests that *Dyb* - and the DGC—may be required for signalling between the neurons and supporting cells and potentially for maintaining the hemolymph-neuron barrier that they provide.

## Materials and methods

### Fly stocks and husbandry

*Drosophila* lines were obtained from the Bloomington *Drosophila* Stock Centre (BDSC) and Vienna *Drosophila* Resource Centre (VDRC). For *Dyb* mutation: *Dyb* ^*EY01099*^ [*y* ^*1*^ *w* ^*67c23*^ *; P[EPgy2]Dyb* ^*EY01099*^ (^#^15494, BDSC)] and *Dyb* ^*11*^ [w*; TI[w[+m*] = TI]Dyb ^*11*^ (^#^78534, BDSC)]. For dystrophin (*Dys*) mutation: *Df(3R)Exel6184* (^#^7663, BDSC). We use Oregon-R, *y* ^*1*^ *w* ^*1*^ (^#^1495, BDSC), and *w* ^*1118*^ (^#^VDRC6000, VDRC) as wild-type references, and as JO defective reference we use an *Fd3F* null line (Newton et al., 2012).

A revertant line of *Dyb* ^*EY01099*^ in which the P element had been precisely excised was generated using the line *Dr* ^*Mio*^; TMS, P{*ry* ^*[+t7.2]*^ = Delta2-3}99B (^#^406, BDSC). This revertant line was named as *Dyb* ^*Ex406*^.

Gal4 driver lines used were: *elav*-Gal4 *(w; elav-gal4;gal80* ^*ts-tub*^ *)*, F-Gal4*, pinta-*Gal4 (*w; pinta-Gal4/TM3,Sb*) and *repo*-Gal4 [*w* ^*1118*^; P{w[+m*]d=GAL4} *repo*/TM3, *Sb* ^*1*^ (^#^7415, BDSC)] were used respectively to target neurons, JO neurons, cap/scolopale cells, ligament cells. These lines were combined with the line UAS-*Dyb* RNAi [*y* ^*1*^ *sc* ^*^ *v* ^*1*^ *sev* ^*21*^ *; P*(*y* ^*+t7.7*^ *v* ^*+t1.8=TRiP.HMS00728*^)*attP2*] (^#^32935, BDSC) for RNAi knock down of *Dyb* in JO cells. The line *attP2* [*y* ^*1*^ *v* ^*1*^ *; P*(*y* ^*+t7.7=CaryP*^) *attP2*] (^#^36303, BDSC) served as control lines for the TRiP collection.

To assess the location of the DGC complex, the Dys-GFP reporter line *y* ^*1*^ w*; Mi ^*PT-GFSTF.0*^ *Dys* ^*MI01893-GFSTF.0*^ */TM3, Sb* ^*1*^ *Ser* ^*1*^ (^#^59782, BDSC) was used. The Dys-GFP line is from a stock collection generated using a Minos Mediated Integration Cassette (MiMIC) (Nagarkar-Jaiswal et al., 2015), and has a GFP module inserted in-frame in the endogenous *Dys* gene. The homozygous flies are viable with no apparent defects. For Iav localisation, the iav-GFP line contains a genomic fusion transgene that rescues the null *iav* mutant (Gong et al., 2004) and has been widely used (Cachero et al., 2011; Newton et al., 2012; Xiang et al., 2022).

### GFP-enhancer transgenic reporter line

A *Dyb* GFP-enhancer transgenic reporter line (*Dyb*-GFP) was designed as follows: the *Dyb* first intron (>10 kb) was found to have a 3-kb region with sequences matching the binding motifs of glial transcription factors, REPO (3 matches, CAATTA) and GCM (ACCCGCA) (Halter et al., 1995). This region was long PCR-amplified from genomic DNA using PFU enzyme and primers designed to include the *BglII* and *XbaI* restriction site. The PCR fragment was cloned into NRE-GreenRabbit (Housden et al., 2012) and selected by kanamycin resistance. After purification, the final vector was validated by restriction enzyme analysis and sequencing. Transformant fly lines were generated by microinjection into syncytial blastoderm embryos of the AttP40 landing site line. Primers are listed in Supplementary Table S1.

### Climbing assay

To assess the role of *Dyb* and DGC in proprioception, we performed a climbing assay. The climbing assays were performed between 13:00 and 15:00 under natural and red light in a climate-controlled room at 21°C. The red light (635 nm) reduces the confounding phototactic component to the behaviour (Sun et al., 2009; Otsuna et al., 2014). Flies were raised in an environmental chamber at 21°C and 25°C. Flies were collected with light CO_2_ anaesthesia the same day of eclosion (day 0). After separating them into female and male groups of 10–20 flies, they were allowed to recover for at least 1 day. Flies were tested on days 2, 10, 20, and 30 after eclosion. On the test day, flies were acclimatised in the behaviour room 2 h before the testing. A climbing tube was made from a 100-ml vertical sealed tube (Kartell) with 5, 10, and 15 cm graduations. Flies were transferred and left 1 min to acclimatise. In the case of the red-light assay, flies were left one extra minute under red light. After banging down, flies were allowed to climb the tube for 10 s. At this point, flies were scored according to their vertical location (1: <5 cm; 2: 5–10 cm; 3: 10–15 cm; 4: >15 cm). The average score constituted the Climbing Index for that batch (*n* = 10 batches per line or cross). All the climbing assays at day 2 were shown as box plots with the mean as middle line and max and min whiskers. Statistical significance was assessed in all comparisons using One-way ANOVA (Kruskal Wallis test), with Dunn’s test for multiple comparisons. For specific comparisons between a defective line and the respective control, we used a Mann-Whitney test.

### Laser Doppler vibrometry

The Laser Doppler Vibrometry assays were carried out in females because they displayed a stable baseline of most transduction parameters up to day 50 (Keder et al., 2020). Flies used for experiments were raised at 25°C. Adult flies aged day 2, 10, 20, and 30 days after eclosion were mounted ventrum-down to the head of a Teflon rod using blue light-cured dental glue. The second segment of the antenna under investigation was glued down to prevent non-auditory background movements, and the other antenna was completely immobilized with the glue. After mounting, flies were oriented in the laser Vibrometer (PSV-400; Polytec, Germany) following previous descriptions (Effertz et al., 2012; Keder et al., 2020). Antenna displacements were measured and digitised to be analysed by the Spike 2 software (Cambridge Electronic Design Ltd., Cambridge, England) using previously described protocols (Keder et al., 2020). Antenna displacements were recorded both before and after the experiment to check physiological integrity. *Dyb* ^*EY01099*^ and control data were tested for normality (Shapiro–Wilk) and equal variance (rejection thresholds set to a *p*-value < 0.05 in both cases) before further statistical analysis. The data that passed the normality and equal variance test were analysed using a two-tailed *t*-test and the remainder with a non-parametric test.

### Immunofluorescence

For antenna staining at the pupal stage, tissue was dissected and fixed in 4% formaldehyde for 10–20 min (Newton et al., 2012), washed in phosphate-buffered saline with 0.3% Triton X-100 (PBT), and then blocked in PBT with 3% bovine serum albumin for 2 h. Antennae were then incubated with primary antibodies in PBT overnight, washed in PBT, and incubated with secondary antibodies for 2h. After washing, tissues were mounted in VectorShield (Vector Laboratories). Antibodies used were goat anti-GFP (1:500; Abcam), mouse anti-Futsch/mAb-22C10 (1:200; Developmental Hybridoma Bank Iowa), mouse anti-REPO (1:500; Developmental Hybridoma Bank Iowa), rabbit anti-DNAH5 [1:2000; (zur Lage et al., 2021)] and mouse anti-NompC [1:250; a gift from X. Liang, Yale University, New Haven, (Liang et al., 2011)]. Secondary antibodies (Thermo Fisher Scientific), DAPI, Alexa Fluor 488 goat anti-rabbit (A-11008), Alexa Fluor goat anti-mouse 488 (A-11001), donkey anti-goat IgG-CFL 488 (sc-362255) and Alexa Fluor 568 goat anti–Mouse (A-11019) were used at a concentration of 1:500. Alexa Fluor 568 phalloidin (A12380, Thermo Fisher Scientific) was used at 1:1000.

### Fluorescence microscopy

Fluorescence images were acquired using a Leica SP8 confocal system, using LAS X Life Science software with the following objectives: 10x (NA >0.70), 20x (NA > 0.90) multi-immersion, and 63x (NA >1.30) oil-immersion. In all cases, images were processed and analysed using LAS X Life Science and FIJI software. Where appropriate, the length of protein staining was measured in three independent experiments. 10 randomly selected cilia in 10 JO z-stacks were analysed for both controls and mutants using the straight line and measure tools from FIJI software.

### Transmission electron microscopy

Whole adult heads of *Dyb* ^*EY01099*^ mutant (*n* = 3) and *yw* control (*n* = 3) flies were removed and prepared following the protocol described previously (Xiang et al., 2022; zur Lage et al., 2018). Sections were examined with a Hitachi 7000 electron microscope, and 124 images were taken. A total of 9 images were longitudinal sections of scolopidium showing the length of scolopidium but not the cell body or horizontal sections higher up in the scolopidium showing ciliary dendrites and neurotubes (see Supplementary Figures S2A–D). These images were used to check cilia defects. The remaining 113 transversal images focussed on the cells bodies were catalogued by their condition: control or mutant, head 1–3 and then blinded for analysis.

The neuronal cell body analysis began by counting the number of inclusions and the number of mitochondria within each cell section using the counter tool from FIJI. During this process, an additional 38 images were excluded for reasons such as a lack of contrast between the cytoplasm and the mitochondria, artefacts, zoomed-in images, and mitochondria, including too many small black dots to count the correct number of inclusions accurately. The number of images does not represent the number of cells analysed, as some images contained up to four cells, so that 162 cell sections (77 images) were analysed. The next step was to measure the area of cells, the total area of mitochondria and the density of mitochondrial inclusions. Both areas were measured manually using the polygon shape tool from FIJI. During this process, an additional 21 images were excluded because they have incomplete cells, so that 89 cell sections (56 images) were measured. Images were analysed using FIJI software.

## Results

### *Dyb* and *Dys* mutant flies exhibit defects in proprioceptive ability

**FIGURE 2:**
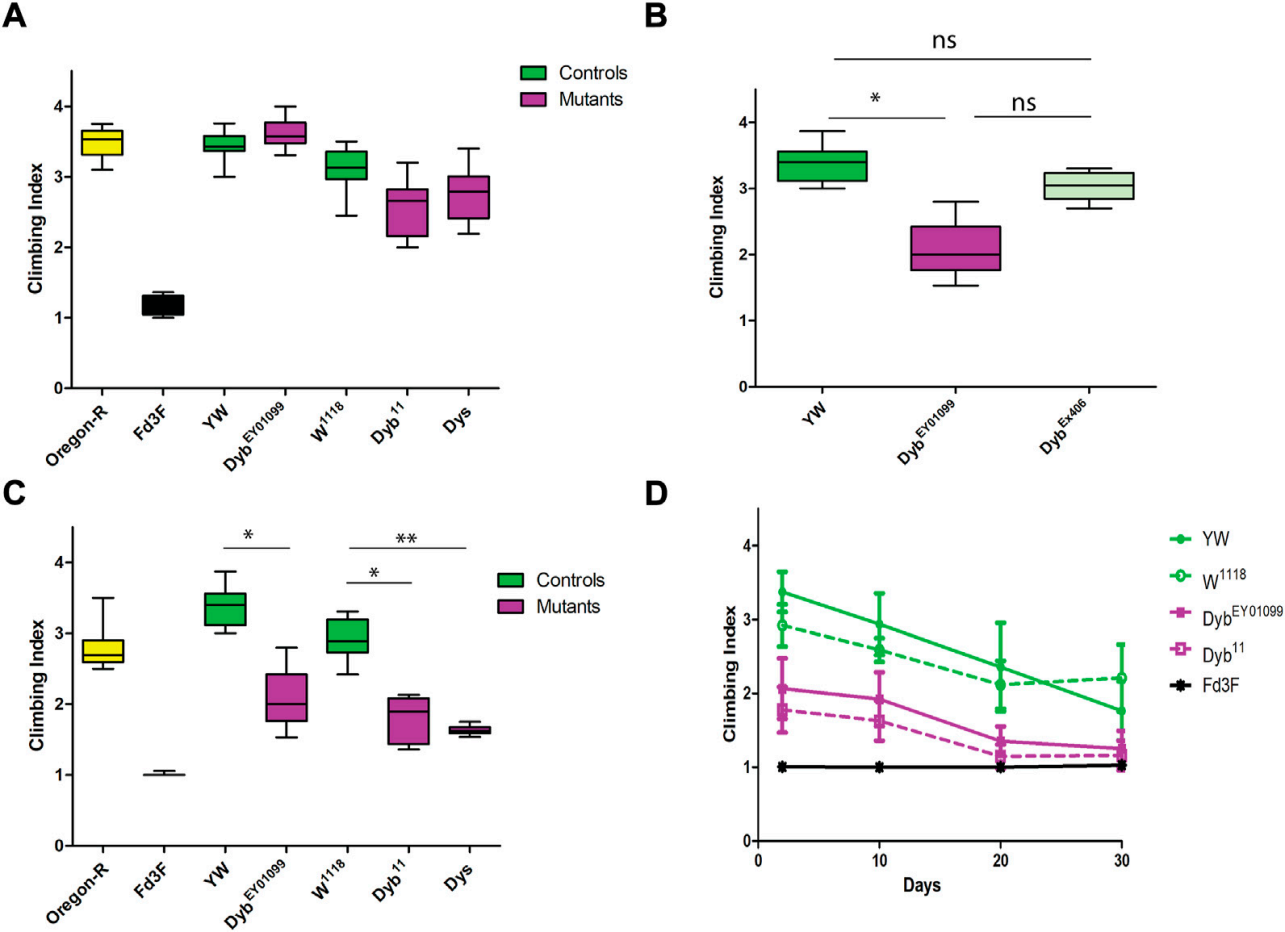
Proprioception ability is defective in DGC mutants and changes over time. Performance of adult females (day 2 after eclosion) in climbing assay. The results of *N* = 10 groups of 15 females are presented as median and interquartile ranges. Flies mutant for *Fd3F* (Black) have poor climbing ability due to non-functional chordotonal neurons, and Oregon-R (yellow) is a wild-type control. **(A)** Performance under natural light conditions showed that *Dyb* ^*EY01099*^, *Dyb* ^*11*^, and *Dys* (magenta) mutant flies exhibit normal climbing behaviour compared to their respective controls (green). **(B)** Performance under red light conditions showed that *Dyb* ^*EY01099*^ and *Dyb* ^*11*^ mutant flies have significantly reduced the climbing ability compared with controls. **(C)** Under red light, *Dyb* ^*Ex406*^ flies (a *Dyb* ^*EY01099*^ revertant line in which the P element impairing the *Dyb* gene has been excised) show significantly improved climbing ability compared with *Dyb* ^*EY01099*^ flies. In addition, there is no significant difference between the control *yw* and *Dyb* ^*Ex406*^. **(D)** Climbing ability of aging flies under red light are presented as mean ± SD. The graph shows the result of *Fd3F* (Black), *Dyb* ^*EY01099*^ (magenta), *Dyb* ^*11*^ (magenta and dashed line), and the respective controls, *yw* (green) and *w* ^*1118*^ (green and dashed line). Under red light, flies with either *Dyb* null mutation, *Dyb* ^*EY01099*^ and *Dyb* ^*11*^, show a mild proprioceptive deficit in a climbing assay relative to the control lines at all young and middle ages (2–20 days after adult eclosion). For climbing assay data, significance was determined by the Kruskal–Wallis test, with Dunn’s test for multiple comparisons. However, only the significant results for DGC mutants (magenta) compared to their respective controls (green) are shown. Significance on plots is signified by asterisks: *p ≤ 0.05; **p ≤ 0.01; ***p ≤ 0.001.

To understand the role of *Dyb* in JO function, we analysed the consequence of genetic loss of function. *Dyb* ^*EY01099*^ is a gene disruption through a P-element insertion, previously used to study *Dyb* in neuron homeostatic plasticity (Younger et al., 2013). *Dyb* ^*11*^ is an amorphic allele previously used to study *Dyb* function in neuromuscular junctions (NMJ) (Jantrapirom et al., 2019). We also examined the role of *Dys,* using a deletion allele (Parks et al., 2004). Flies homozygous for all these alleles are viable and show no gross anatomical differences from controls. To test proprioception, we performed climbing assays. We examined the ability of flies to climb 15 cm in 10 s under natural and red light (635 nm) conditions. Initially, we observed the flies on day 2 after eclosion. When tested under natural light conditions, mutant flies exhibited no significant difference in climbing ability from controls (Figure 2A, Supplementary Table S2). Since the tendency for flies to climb upwards depends on both proprioceptive and visual stimuli, we also tested under red light conditions. While *Drosophila* can likely detect red light to a limited extent (Sharkey et al., 2020), this simulates conducting the assay in the dark, which has been shown to reveal proprioceptive defects in mutants that otherwise appear normal under light conditions (Sun et al., 2009). In red light, all three mutants showed a significant reduction in the climbing ability, with a stronger effect for *Dys* than for *Dyb* (Figure 2B, Supplementary Table S3). To confirm that the effect observed in the *Dyb* ^EY01099^ line is caused by the disruption of *Dyb*, we precisely excised the P-element to generate the revertant line *Dyb* ^*Ex406*^. The results under red light show an improvement in the climbing ability compared with *Dyb* ^EY01099^ (Figure 2C).

The climbing defect is revealed only upon removing substantial visual stimulus. This supports the conclusion that the flies exhibit a proprioceptive defect due to JO malfunction (that can be compensated by visual sensory input) rather than a motor defect. Also these results corroborate recent findings that a visually guided leg-movement control exists in *Drosophila* (Cruz et al., 2021). In contrast, a mutation that entirely and specifically abolishes chordotonal organ function, *Fd3F*, results in an extreme impairment of climbing in both light and dark (Cachero et al., 2011; Newton et al., 2012) (Figure 2). Thus, impaired function of all chordotonal organs (in JO, legs, elsewhere) results in a climbing deficit that cannot be compensated by visual cues. In contrast, as *Dyb* impairment is only revealed when visual input is largely removed, this may indicate either a milder defect in chordotonal organ function, or a defect in JO specifically (Sun et al., 2009).

MD is characterised by a worsening of symptoms over time. We assessed proprioception ability across the *Drosophila* life course by performing the climbing assay at different ages, including days 2, 10, 20, and 30 after eclosion. Under red light conditions, both *Dyb* null fly lines showed a moderate proprioceptive deficit in a climbing assay relative to the control lines at all young and moderate ages (2–20 days after adult eclosion). Again, this defect is only apparent when visual cues are removed during the assay (Figure 2D). The decline observed over time means that *Dyb* mutant flies exhibit premature aging with respect to proprioceptive ability.

### *Dyb* mutant flies show hearing loss and auditory homeostasis deficit

**FIGURE 3:**
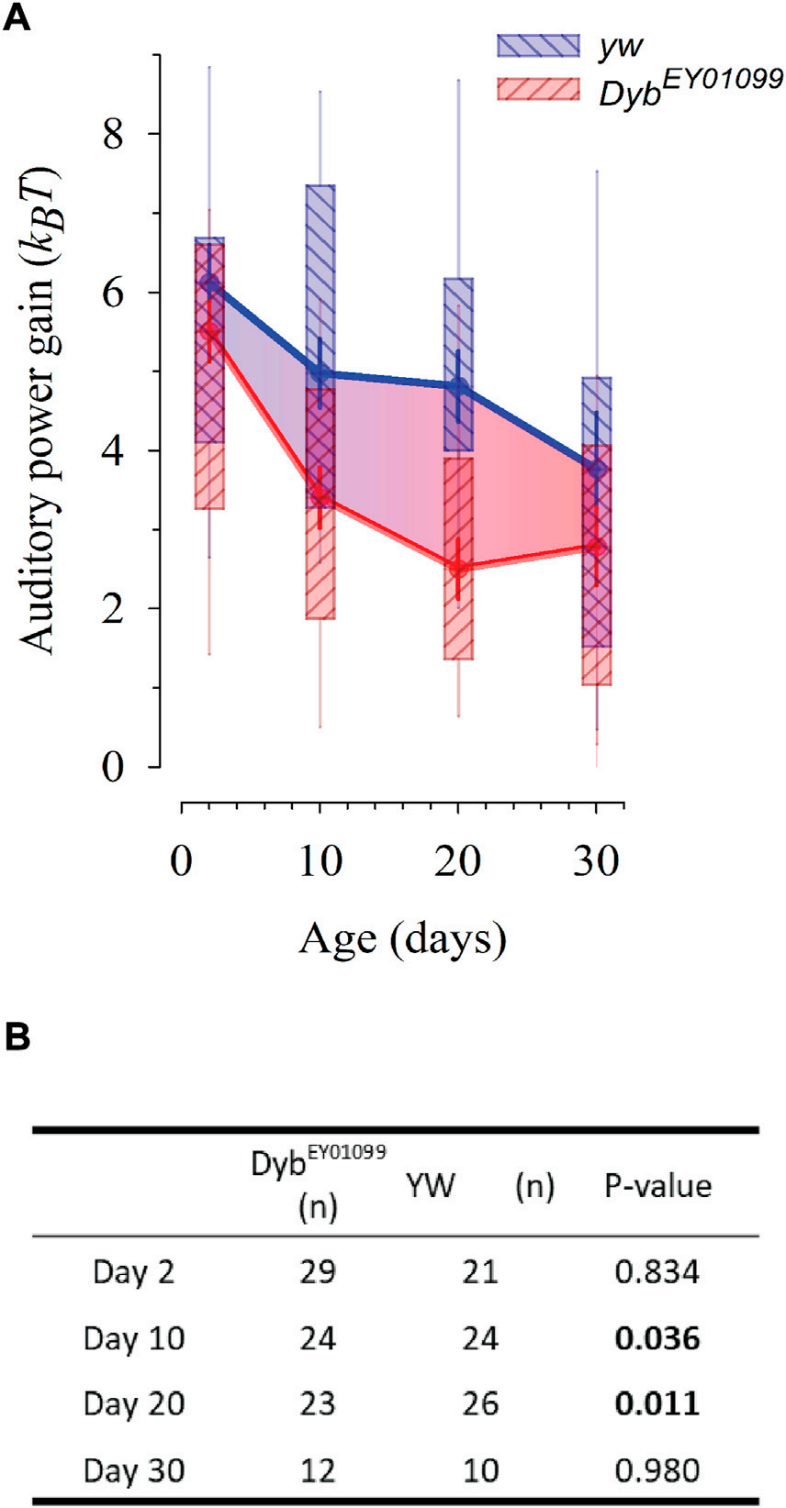
*Dyb* ^*EY01099*^ mutant flies present with progressive, early onset hearing loss. The life course of auditory power gain (ΔKBT, the fly’s ‘cochlear amplifier’) is affected in *Dyb* ^*EY01099*^. Data presented as median ± SEM. **(A)** The loss of auditory amplification starts earlier, and is steeper, in *Dyb* ^*EY01099*^mutants as compared to their *yw* controls. While at day 2, amplification in *Dyb^EY01099^* mutants is statistically identical to that of *yw* controls (*p* = 0.834), it falls significantly below control levels at day 10 (*p* = 0.036) and day 20 (*p* = 0.011). At day 30, the slower, yet constant hearing decline of controls has caught up with the accelerated hearing loss seen in *Dyb* ^*EY01099*^, and no more statistical difference is observed (*p* = 0.980). The box ends demarcate the 25th and 75th percentiles; fine error bars define the 10th and 90th percentiles. Symbols reflect the medians and thick error bars the standard error of the median. **(B)** Statistical data for auditory power gains in *Dyb* ^*EY01099*^and *yw* controls across ages. The power reduction seen in middle-aged flies is equivalent to a moderate to severe hearing loss condition in humans and suggestive of a disturbed auditory homeostasis. Test: One-way ANOVA, with pairwise multiple comparisons using the Holm-Sidak method.

JO auditory neurons are active mechanosensors that inject energy into sound-induced receiver motion, equivalently to hair cells in the vertebrate inner ear (Albert and Kozlov, 2016). The auditory function of JO can be assessed by measuring the free mechanical fluctuations of unstimulated antennae through Laser Doppler Vibrometry. The fly’s active hearing can be quantified by three principal parameters: the receiver’s best frequency (f0, around 200 Hz in a healthy, active system), their frequency selectivity (Q) and their energy gain (measured as K_B_T). *Dyb* ^*EY01099*^ antennae show alterations in these essential parameters. At day 2 and day 30, *Dyb* ^*EY01099*^ flies did not differ significantly from their *yw* controls; however, at days 10 and 20 *Dyb* ^*EY01099*^ mutant flies showed significant reductions in energy gain (Figure 3). This reduction suggests a breakdown of the active hearing process and resulting hearing loss.

**FIGURE 4:**
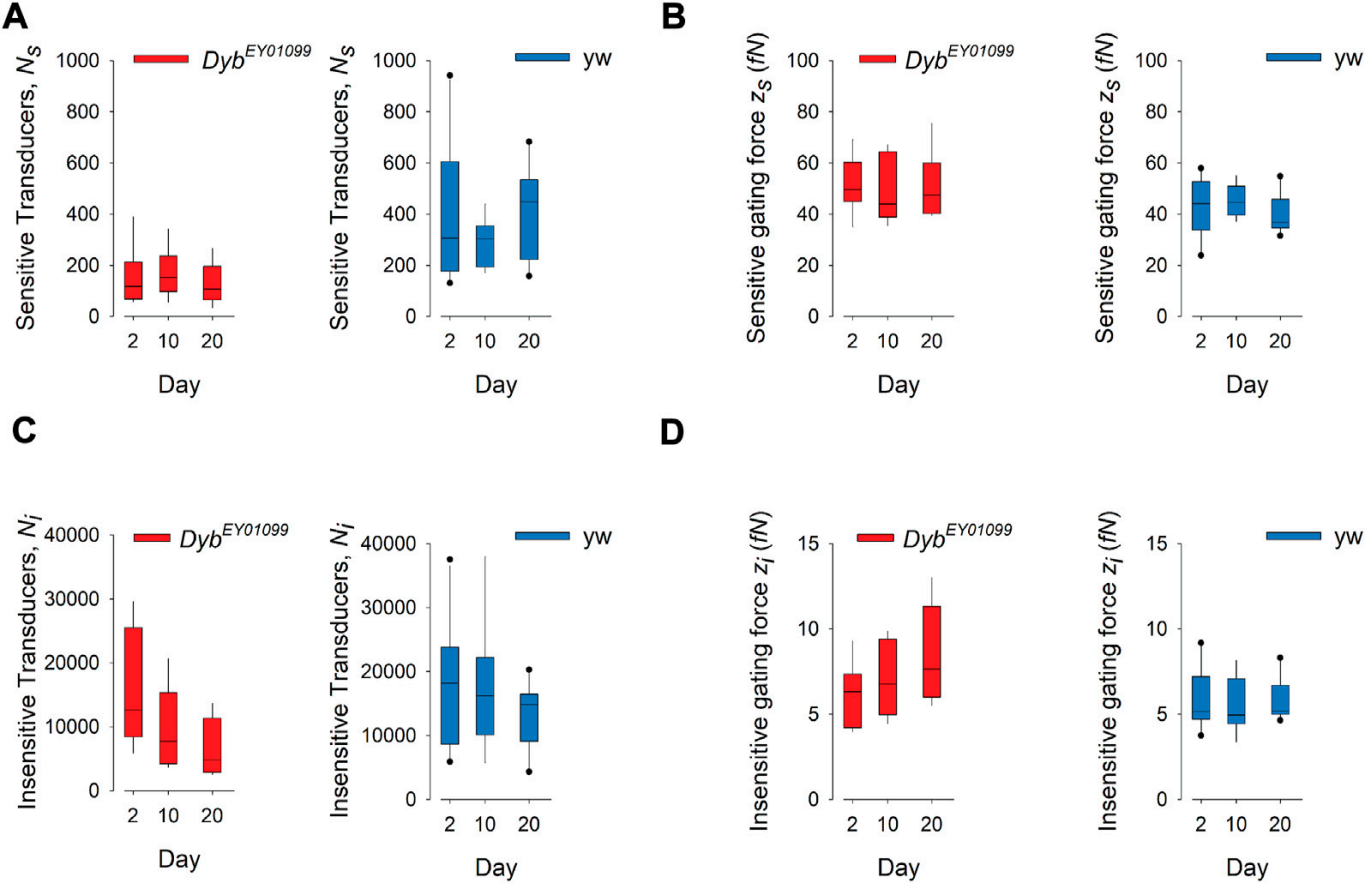
Mechanotransduction in *Dyb* ^*EY01099*^ mutants is impaired in a submodality-specific way. **(A)** The number (Ns) of sensitive (auditory) transducers is significantly (*p* < 0.05) reduced in *Dyb* ^EY01099^ (left) as compared to their *yw* controls (right) across all ages tested (day 2: *p* = 0.026; day 10: *p* = 0.0115, day 20: *p* = 0.000752). No progressive, age-dependent loss of sensitive transducers is observed in either mutants or controls (*p* = 0.632 and *p* = 0.470, respectively; Kruskal–Wallis One Way ANOVA on Ranks; *n* = 10 each). **(B)** The gating force (z_s_) of sensitive transducers is significantly enhanced in *Dyb* ^EY01099^ (left) as compared to their *yw* controls (right) at day 20 but unchanged before (day 2: *p* = 0.143; day 10: *p* = 0.36, day 20: *p* = 0.028). No significant progressive, age-dependent change of gating force is observed (mutants: *p* = 0.819; controls: 0.276). **(C)** The number (Ni) of insensitive (wind/gravity) transducers is significantly (*p* < 0.05) reduced in *Dyb* ^EY01099^ (left) as compared to their *yw* controls (right) across all ages tested (day 2: *p* = 0.629; day 10: *p* = 0.0603, day 20: *p* = 0.0079). A progressive, age-dependent loss of insensitive transducers is observed in *Dyb* ^EY01099^, but not in *yw* controls (*p* = 0.046 and *p* = 0.459, respectively; Kruskal–Wallis One Way ANOVA on Ranks; n = 10 each). **(D)** The gating force (z_i_) of insensitive transducers is significantly enhanced in *Dyb* ^EY01099^ (left) as compared to their *yw* controls (right) at day20 but unchanged before (day 2: *p* = 0.74; day 10: *p* = 0.115, day 20: *p* = 0.0171; *t*-test or Mann-Whitney Rank Sum test). No significant progressive, age-dependent change of insensitive gating force is observed (mutants: *p* = 0.235; controls: *p* = 0.787). In all box plots, box ends demarcate the 25th and 75th percentiles; error bars define the 10th and 90th percentiles, with a horizontal line at the median.

To probe auditory function in more detail, we quantified the mechanical signatures of auditory mechanotransduction in response to force-step actuation of the JO in females of different ages (Figure 4). The direct gating of mechanotransducer channels produces characteristic nonlinearities—more specifically, it introduces characteristic stiffness drops into the mechanics of the antennal sound receiver. These “gating compliances” (compliance being the inverse of stiffness) can be quantified with a simple gating spring model, thus allowing for extracting the number—and properties—of populations of mechanotransducers present in JO. Two such populations have previously been identified: a “sensitive” population, which mediates hearing, and an “insensitive” population, mediating the sensation of wind and gravity (Effertz et al., 2012).

The number of sensitive transducers (*N*_*s*_) is significantly reduced in *Dyb* mutants of all ages, compared to their respective *yw* controls (Figure 4A), suggesting a *Dyb*-related, but age-independent, loss of auditory function. Interestingly, the number of insensitive, i.e., wind-gravity related transducers (*N*_*i*_) falls in an age-dependent manner in *Dyb* mutants, while they remain stable in the *yw* controls (Figure 4C). This finding suggests that there is a progressive, age-dependent loss of mechanosensory function in *Dyb* mutants, which specifically affects the “insensitive” transducer channels, contributing to the sensory submodalities for balance, wind, and gravity.

The operation of mechanosensory neurons in the *Drosophila* JO relies on complex interactions between 1) the number of functional transducer channels, 2) their specific properties (e.g. their single channel gating forces), and 3) motor proteins (e.g., dyneins) that act in series with the transducers to facilitate adaptation and amplification (power gain). In-depth quantifications of auditory ageing have revealed that—especially in females—the performance of the *Drosophila* ear (including its power gain and tuning) (Keder et al., 2020), and the numbers of functional transducers (both sensitive and insensitive) can stay roughly constant up to an age of 50 days. Moreover, homeostatic stabilisation mechanisms exist (Keder et al., 2020), which can partly compensate for a loss of transducers by adjusting their molecular properties (e.g., increasing their single channel gating forces). This process is also visible in the progressive, early-onset loss of insensitive transducers seen in *Dyb* ^EY01099^ flies (Figures 4C,D). The non-progressive, age-independent loss of sensitive transducers that characterises the *Dyb* ^*EY01099*^ mutant phenotype, in contrast, points to a developmental deficit (possibly linked to a mislocalisation of mechanosensory NompC TRP channels, see below at Figure 8), which is only partly compensated by an increase of the corresponding single channel gating forces (Figures 4A,B). A homeostatic interrelation between channel numbers and their respective gating forces has been reported before (Keder et al., 2020). Together with the reported environmental dependencies of these parameters (as for example developmental temperatures (Boyd-Gibbins et al., 2021), these dynamic homeostatic conditions might also explain the variance of reported values observed between different studies (Effertz et al., 2012; Keder et al., 2020; Boyd-Gibbins et al., 2021).

Together with the progressive loss of auditory amplification seen in *Dyb* ^*EY01099*^ ears (Figures 3A,B), the biophysical data of JO reveal developmentally weakened auditory transducer apparatuses after loss of *Dyb* function, with subsequent failures of the ear’s homeostatic maintenance. The extent of the resulting decay of auditory amplification seen in *Dyb* mutants at days 10 and 20 - despite a partial compensation on the level of ion channels - suggests that other molecular modules of mechanotransducer function, e.g., dynein-based motor molecules, are impaired as well.

### *Dyb* is required in ligament support cells and possibly neurons for Johnston’s organ function

**FIGURE 5:**
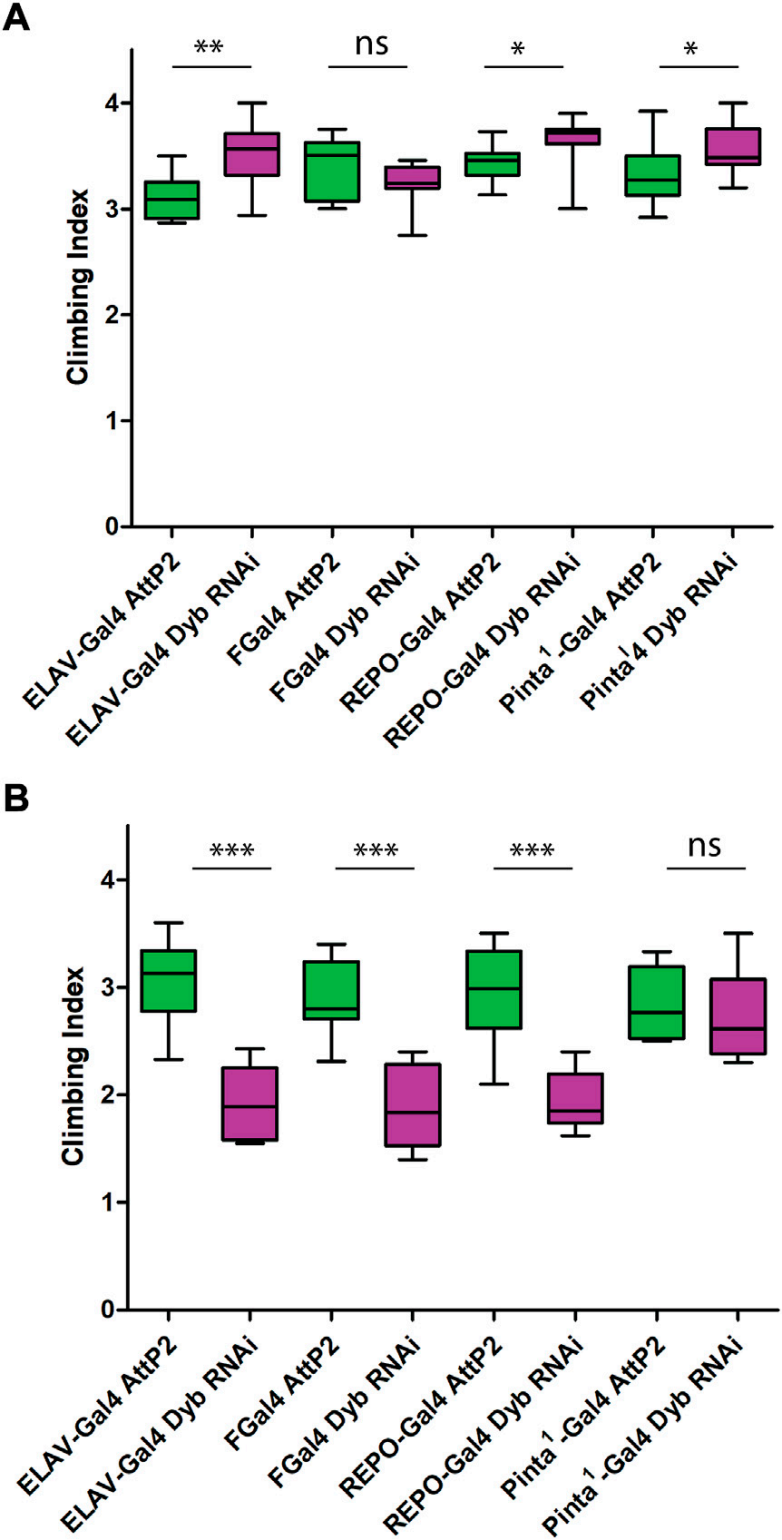
Proprioception ability is defective in cell type-specific impairment of *Dyb* function. Performance of adult females (day 2 after eclosion) in climbing assay under natural light conditions **(A)** and red light conditions **(B)**. The results of *N* = 10 groups of 15 females presented as median and interquartile ranges. In the graph, we show the results of four *Dyb* cell type-specific knockdown; *elav*-Gal4 UAS-*Dyb*-RNAi (neurons), FGal4 UAS-*Dyb*-RNAi (JO neurons), *pinta*-Gal4UAS-*Dyb*-RNAi (cap/scolopale cells) and *repo*-Gal4 UAS-*Dyb*-RNAi (ligament cells). For climbing assay data knockdown (magenta) was compared again to their respective controls (green) using a Mann-Whitney test. **(A)** Under natural light conditions, flies of *elav*-Gal4 UAS-*Dyb*-RNAi, *repo*-Gal4 UAS-*Dyb*-RNAi and *pinta*-Gal4 UAS-*Dyb*-RNAi showed a significant increase in the climbing ability compared with their control (*p*-value = 0.0021, 0.01 and 0.0448, respectively). **(B)** Under red light conditions, flies with *Dyb* knocked down in neurons and ligament cells showed significantly reduced climbing ability compared with controls (***p-value < 0.001). *Pinta*-Gal4 UAS-*Dyb*-RNAi does not show any difference. Significance on plots is signified by asterisks: *p ≤ 0.05; **p ≤ 0.01; ***p ≤ 0.001.

To find further evidence of a direct sensory deficit associated with the JO and to assess the cellular location of *Dyb* function within JO, we used the Gal4/UAS system to knock down *Dyb* activity in specific cell types. We used Gal4 lines for all neurons (*elav*-Gal4), JO neurons (F-Gal4), cap/scolopale supporting cells (*pinta*-Gal4) and ligament/glial cells (*repo*-Gal4). Under natural light conditions, UAS-*Dyb*-RNAi driven by each driver showed no loss of climbing ability compared with their controls (in fact, all showed a relative increase) (Figure 5A). However, under red light, knockdown driven by *elav*-Gal4 and *repo*-Gal4, but not *pinta*-Gal4, significantly reduced climbing ability compared with controls (Figure 5B). This suggests that *dyb* function is required in neurons and ligament cells, but not cap or scolopale cells.

### *Dyb* is expressed in the ligament cells

**FIGURE 6:**
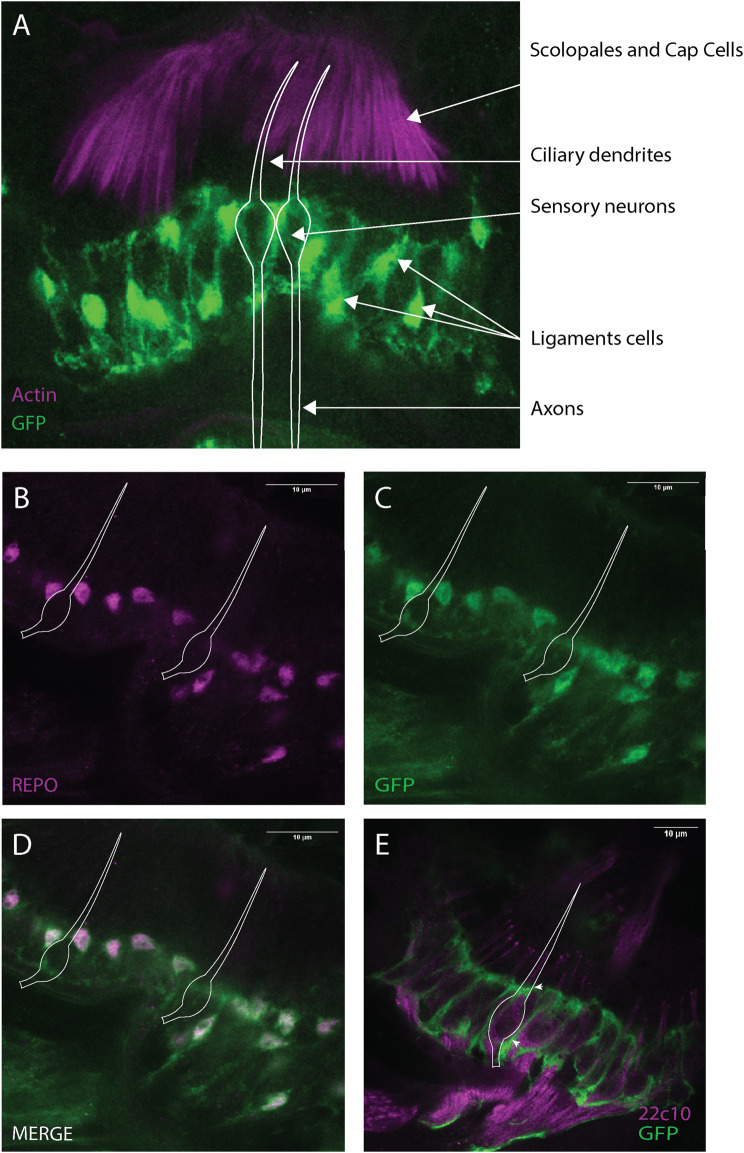
*Dyb* is expressed in JO, mainly in the ligament cells. Immunofluorescence performed on pupal antennae from the *Dyb*-GFP. **(A)** Immunofluorescence performed using phalloidin (marker of actin-rich scolopale, magenta) and anti-GFP antibodies (green). The expression of the *Dyb*-GFP reporter gene resembles the location of ligament cells ensheathing the neuronal cell bodies. The cytoplasmic GFP reveals the glia-like nature of these supporting cells. **(B–D)** Immunofluorescence performed using anti-REPO (magenta, a nuclear glia cell marker located in ligament cells) and anti-GFP antibodies (green). The co-labelling with REPO confirms *Dyb* expression in the ligament cells. **(E)** Immunofluorescence with sensory neuron marker anti-Futsch (mAb22C10, magenta), and anti-GFP (green) antibodies showing the GFP-expressing ligament cells ensheathing the neuronal cell bodies. The white diagram in all the images represents the approximate locations of example sensory neurons. Scale bars: 10 μm.

To characterise *Dyb* expression in JO, we constructed a reporter line with GFP under the control of a DNA element from the first intron of *Dyb*. Immunofluorescence with phalloidin as a counterstain (marking the actin-rich scolopale and cap structures) revealed *Dyb*-GFP reporter expression within pupal JO in the region of the neuronal cell bodies (Figure 6A). However, co-labelling with a glial marker, anti-Repo, revealed that this expression was in ligament cells rather than in the neurons themselves (Figures 6B–D). The imaging revealed that these supporting cells have glial-like projections that ensheath the neuronal cell bodies, marked with anti-Futsch (Figure 6E). In conclusion, these results confirm the expression of *Dyb* in the ligament cells, a major site of *Dyb* function revealed above. As this is a reporter gene construct, it does not rule out the possibility that *Dyb* is also expressed in other JO cell types.

### Dystrophin protein localises to support cells, including the ligament cell

**FIGURE 7:**
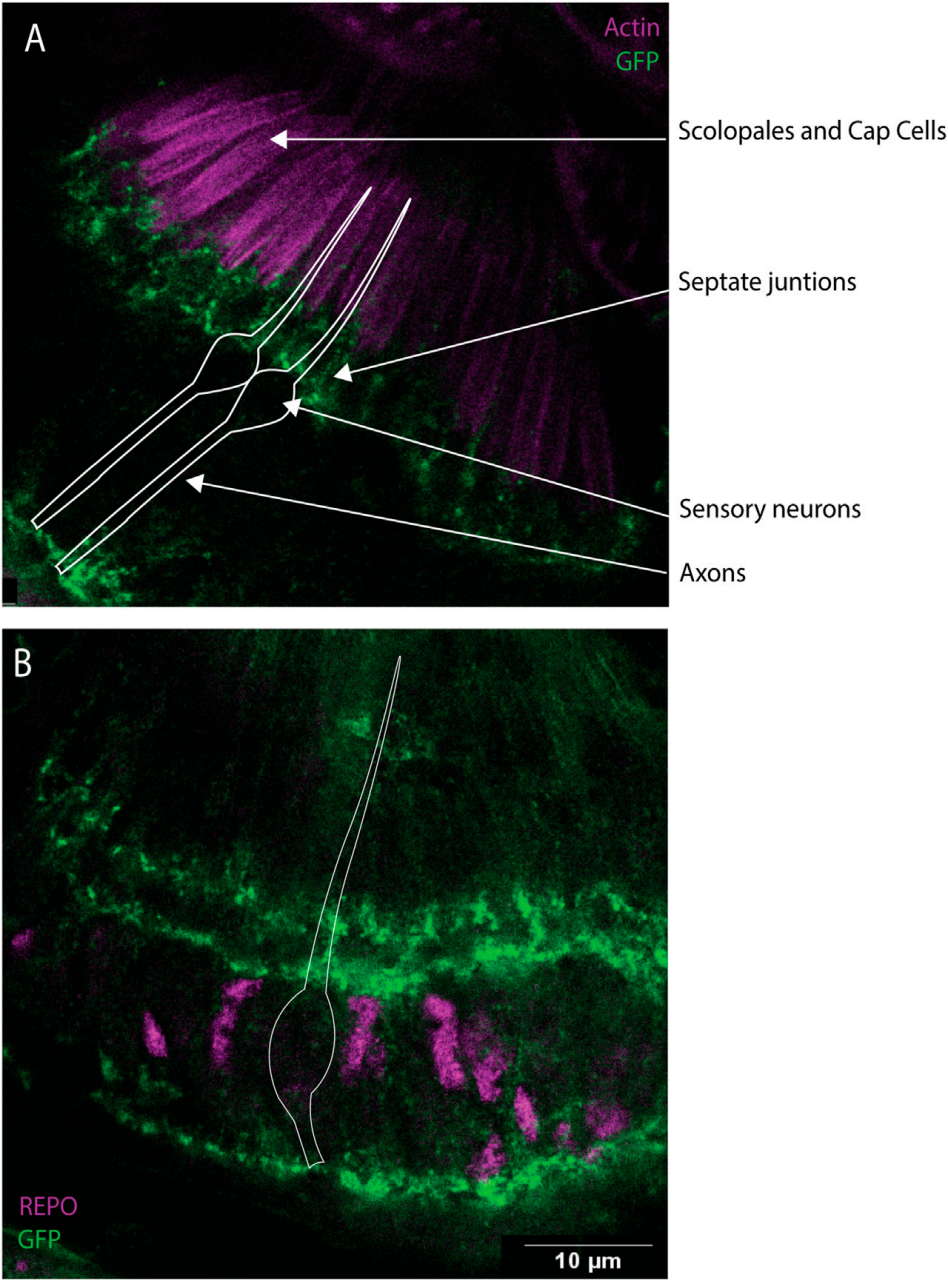
Dys expression in JO, mainly in the scolopale and ligament cells. Immunofluorescence performed on pupal antennae from fly line Dyb-GFP. **(A)** Immunofluorescence performed using anti-GFP antibodies (green) and phalloidin (magenta), which marks the actin bundles present in the scolopale and cap cells. This identified the expression of *Dys* protein in the basal part of the scolopale cells. **(B)** Immunostaining performed using anti-REPO (magenta) and anti-GFP antibodies (green). Dys expression is concentrated in three specific locations, the basal part of the scolopale cell, the top part of the ligament cells at sites predicted to be in contact with the scolopale cells (septate junctions), and in the basal area of the ligament cells where they connect with the JO cuticle. The white diagram represents the shape of the sensory neuron. Scale bars: 10 μm.

Dyb protein has been previously associated with the DGC complex in the larval brain (Jantrapirom et al., 2019). In addition, according to scRNA-seq data in FlyCellAtlas, *Dys* is expressed in 36% of the JO neurons and 54% of the antennal glia cells (Li et al., 2022). To identify the location of the DGC in JO, we analysed flies containing a transgene that tags endogenous Dystrophin protein. Immunostaining using anti-GFP and phalloidin or anti-REPO showed that Dystrophin is present in a highly localised pattern within support cells (Figures 7A,B). Dystrophin (and by inference the DGC) is concentrated in three specific locations within JO supporting cells: the basal part of the scolopale cell, the distal part of the ligament cells at the contacts with scolopale cells (containing septate junctions), and in the basal area of the ligament cells where they connect with the JO cuticle. This suggests that DGC functions in maintaining structural or functional integrity of contacts between JO supporting cells.

### Johnston’s organ structure is largely intact in *Dyb* mutant flies, but there are changes in neuronal mitochondria and in NompC channel localisation

To explore whether the absence of *Dyb* causes structural changes in JO, we began by assessing ligament cell numbers. Immunostaining pupal antennae with anti-REPO and DAPI and cell counting showed a slight reduction in the number of cells in the *Dyb* ^*11*^ mutant JO compared with the control, but no significant differences were found (Supplementary Figure S1). Similarly, no difference in neuronal cell number was observed.

Transmission electron microscopy (TEM) was performed to examine JO ultrastructure. In the antennae of *Dyb* ^*EY01099*^ mutant adults, the structure of the sensory neurons appeared grossly normal, including well-formed mechanosensory cilia, with correct morphology and axonemal structure, a typical axonemal structure of 9 + 0 microtubule doublets, and with the presence of both outer and inner dynein arms (Supplementary Figures S2A,B). Longitudinal sections through scolopales show a similar cilium structure with the distal basal bodies and ciliary dilations (Supplementary Figures S2C,D). Similarly, no gross defects could be observed in supporting cell structures (e.g., scolopale structure, attachment of ligament to cuticle), and the septate junction is present in both genotypes at an early age (Supplementary Figures S2E,F). Also, no obvious differences were found in the cell bodies transverse sections (Supplementary Figures S2G,H). The mean area of individual neuronal cross-sections of the *yw* and *Dyb* ^*EY01099*^ groups was 12.40 and 11.06 μm^2^, respectively (Supplementary Figure S2I).

We examined neuronal mitochondria in more detail. The number of mitochondria cross-sections per neuron was not significantly different between the *Dyb* ^*EY01099*^ and *yw* groups (Supplementary Figure S3A), and although the mean total mitochondria area per neuronal section appeared higher in *Dyb* ^*EY01099*^ sections, these data were not significant (Supplementary Figure S3B). In addition, we observed frequent small dense particle inclusions within the mutant mitochondria Supplementary Figures S3C,D). A nested *t*-test revealed the number of inclusions was significantly greater in *Dyb* ^*EY01099*^ neuronal mitochondria compared to *yw* (nested *t*-test: t4 = 4.569, F1,4 = 20.88, *p* = 0.0103) (Supplementary Figure 3E). The density of inclusions in mitochondria per cell section was not significantly different, but a trend was observed (Supplementary Figure 3F). These observations suggest that *Dyb* mutation could, directly or indirectly, cause changes in mitochondria that may be consistent with stress or functional impairment.

**FIGURE 8:**
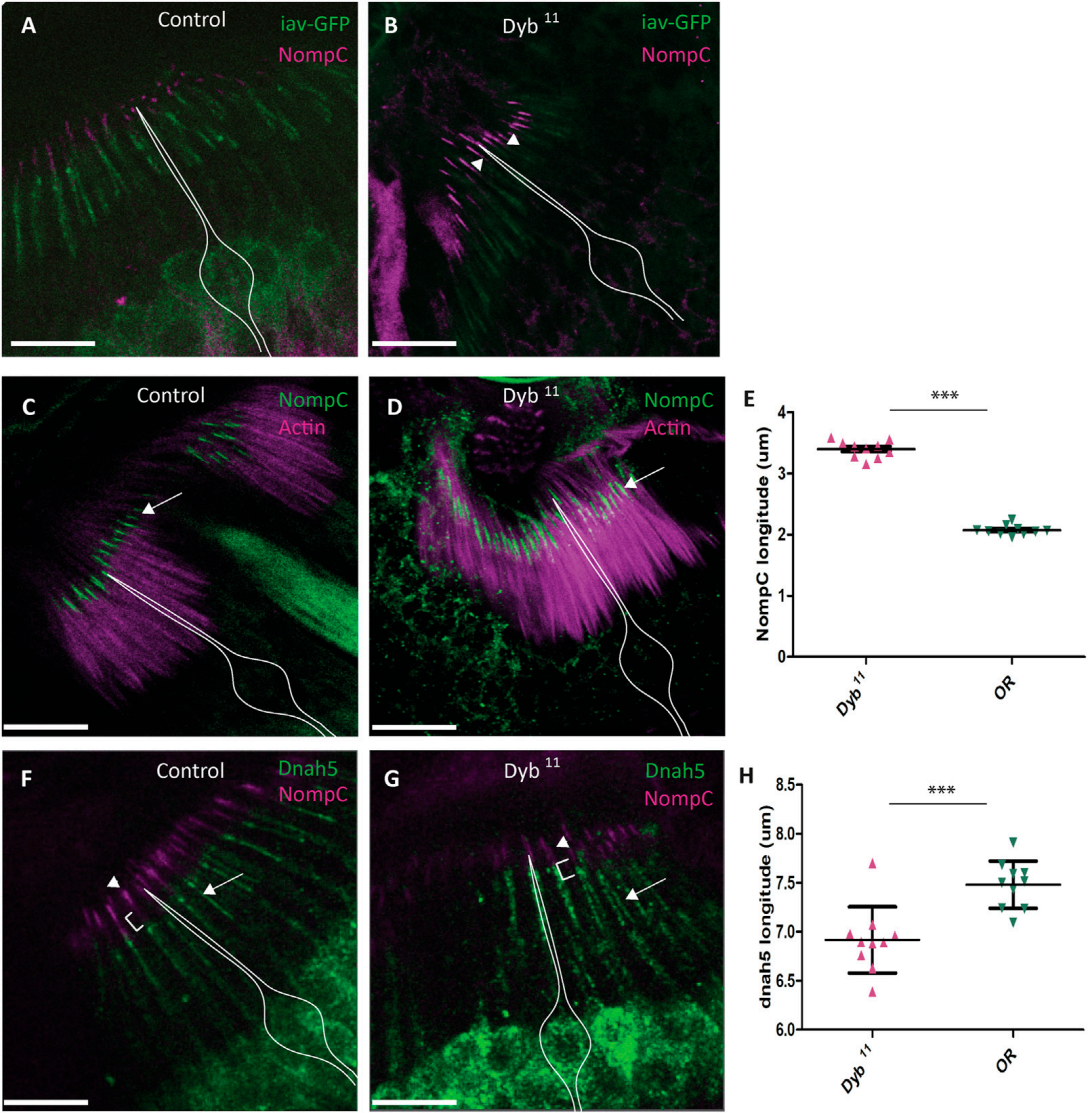
Location and distribution of dynein heavy chain Dnah5 and TRP channels, NompC and Iav, in *Dyb* ^*11*^ mutant JO neurons. Confocal images of immunofluorescence in JO at pupal stage. **(A)** In control JO (OR), TRP channels Iav (green) and NompC (magenta) localise to separate zones of the sensory neuron ciliary dendrites. **(B)** In the *Dyb* ^*11*^ mutant JO, a diffuse distribution of NompC (magenta), causes it to overlap with Iav (green) in the cilia dendrites (arrowhead). Scale bar: 10 μm. The white outline represents the location of sensory neurons. **(C)** TRP channel NompC (green) localise to the extreme tips of the cilia dendrites in the OR control (white arrow). The actin marker identifies the scolopale cell. **(D)** In *Dyb* ^*11*^, NompC is most diffusely located in the ciliary dendrites (white arrow). The actin marker identifies the scolopale cell. **(E)** Length of ciliary extent of NompC in *Dyb* ^*11*^ mutant and control antennae. In three independent experiments, NompC extent was measured in 10 cilia from each of 10 JO (100 cilia) for both controls and *Dyb* ^*11*^. In the graph, each dot represents the mean of 10 cilia from one antenna. Comparison was performed by Mann Whitney test. Length of ciliary extent of NompC in *Dyb* ^*11*^ is significantly greater (*p*-value = *p* < 0.0001). **(F)** Axonemal dynein heavy chain Dnah5 (green, white arrow) and NompC (magenta, arrowhead) localise to separate zones in the cilia dendrites in OR control. **(G)** In *Dyb* ^*11*^, NompC (magenta, arrowhead) is diffusely located in the cilia dendrites, and it extends closer to Dnah5 (green, white arrow). **(H)** Length of ciliary extent of Dnah5 in *Dyb* ^*11*^ is significantly shorter than in the control (*p*-value = *p* < 0.0001). Experiment performed as described in **(E)**.

Several transient receptor potential (TRP) channels are required for correct auditory transduction (Gopfert et al., 2006; Effertz et al., 2012; Zanini and Gopfert, 2014). Although the sensory cilia appeared intact ultrastructurally, the investigation of auditory function above suggested defects in mechanotransduction, perhaps linked to abnormal function of NompC, the TRPN channel homologue required for mechanosensation. To investigate the structure further we determined the ciliary localisation of two TRP channels. In wild type flies, the TRPV channel homologue, Iav, is located in a proximal region of the cilium. In *Dyb* ^*11*^ mutant pupal antennae, Iav localisation in the proximal cilium appears similar to that of the control antennae (Figures 8A,B). In contrast, NompC, is usually located in the distal section of the cilium, where it inserts into the extracellular cap structure. In *Dyb* ^*11*^ mutant pupal antennae, NompC protein is still located distally, but in a broader region of the distal cilium than normal (Figures 8C–E). Localisation appears to extend further proximally, such that it atypically overlaps with Iav (Figures 8A,B). Mechanotransduction also requires the presence of axonemal dynein motors in the proximal cilium (Karak et al., 2015). As a marker of these motors, we used antibodies that detect the outer dynein heavy chain homologue, Dnah5 (zur Lage et al., 2021). In *Dyb* ^*11*^ mutant pupal antennae, Dnah5 protein localised to the proximal cilium (Figures 8F,G) but the Dnah5-positive section of cilium is shorter than in controls (Figure 8H).

In conclusion, in *Dyb* mutants, the gross structure and molecular composition of the chordotonal neurons and their ciliary dendrites appear largely intact, but mislocalisation of NompC and Dnah5 suggest an underlying ciliary compartmentation defect, which may explain the changes in mechanotransduction parameters observed above.

## Discussion

Although it is known that α-dystrobrevin and the DGC are expressed in the mammalian inner ear, their functions in the ear are unclear besides the observation that *DTNA* mutations have been linked to MD. Our results in *Drosophila* reveal that DGC components Dyb and Dystrophin are expressed in supporting cells of the fly’s inner ear and that Dyb is required for correct JO function in hearing and proprioception. Two physiological defects are observed. The first is developmental (present from eclosion of the adult) related to an age-independent constant reduction of sensitive (auditory) transducer channels. This is possibly related to the observed mislocalisation of auditory NompC channels. The second defect is progressive, related to an impairment in homeostasis of JO function. We speculate that this impairment can be observed in the mitochondrial phenotypes that may reflect physiological stress of the sensory neurons. However, given the localisation of Dyb and Dys proteins to support cells, we suggest these neuronal defects are indirect consequences of a primary role in support cells. For example, in mammals, hair cells are susceptible to many kinds of stress/perturbation, including acoustic overstimulation, ototoxic drugs, or changes in the inner ear associated with normal ageing (Op de Beeck et al., 2011). Supporting cells provide protective mechanisms (May et al., 2013), but if these are overwhelmed or impeded, the hair cells exhibit an overt phenotype. Thus we hypothesise that the DGC impairment affects the supporting cells restricting their protective role and could interfere with the ion composition of the endolymph. This role may be related to functions in securing the hemolymph-neuron barrier and receptor lymph homeostasis within the scolopales. We discuss these findings and their implications for mammalian inner ear function and MD.

### Possible cellular functions of dystrophin-glycoprotein complex in Johnston’s organ

*Dyb* function, and the loss thereof, affects the operation of sensory transducer channels in JO neurons, as evident from alterations in principal parameters of ciliary mechanotransduction and subtle mislocalisations of some of its key mechanotransduction proteins. However, we find prominent expression of Dyb and Dystrophin in supporting cells, suggesting that these are the primary location of DGC function. The expression patterns of these genes in the mammalian inner ear are unclear, but the evidence suggests expression in supporting cells too. In humans and mice, Dystrobrevin and DGC are expressed in different inner ear supporting cells (Ishiyama et al., 2018; Gu et al., 2021), including Reissner, Spindle and Root cells (Orvis et al., 2021). Protein locations have been only validated with immunostaining in vestibular supporting cells but these suggest that Dystrobrevin is located in the basal part of supporting cells in cristae (Requena et al., 2015), while Dystrophin may be localised to hair cells (Dodson et al., 1995). Recent RNAseq data from the mouse organ of Corti suggest that Dystrobrevin and Dystrophin are transcribed in all the inner ear cell types (Kolla et al., 2020). Further studies are required to clarify the location of the DGC in the mammalian ear.

In JO, a major function of the ligament cell type is thought to be structural, anchoring the scolopidia to apodemes of the antennal cuticle (Todi et al., 2004). Dystrophin-GFP localised to the ligament cell projections that may contact the cuticle. However, no attachment defect was seen in the *Dyb* ^EY01099^ JO by TEM or immunostaining. It is possible that dystrophin/DGC has a role at this location that is independent of Dyb or that any role of Dyb protein at the ligament-cuticle interface is more subtle and only revealed under specific conditions. Indeed, in the heart of *Dtna* knockout mice it was found that the DGC complex localisation is normal but the tissue is weaker and highly susceptible to membrane damage with cardiac stress (Strakova et al., 2014). Defects after stress conditions have also been documented in the *mdx* (mutant dystrophin) mouse inner ear, showing hearing loss after noise exposure (Michel et al., 2000).

Apart from structural roles, major physiological roles of support cells in both *Drosophila* and mammalian ears are the formation of a blood-brain barrier and associated maintenance of the receptor lymph bathing the mechanosensory cells. It is thought that α-Dystrobrevin’s role in the DGC is linked to recruitment and organisation of signalling molecules in DGC-associated cell junctions in NMJs and synapses *via* syntrophins. It has been described that Dystroglycan-Dystrophin-Syntrophins are involved in the gene expression regulation required to adapt neuron homeostasis under stress and dystrophic conditions (Kucherenko et al., 2011). This regulation is carried out through microRNAs like miR-956, miR-980, and miR-252 (Marrone et al., 2011). In addition, the Ca-activated K channel encoded by *slowpoke* and *dyschronic* (*dysc*), which encodes the orthologue of deafness-associated protein Whirlin/Dfnb31, influences diverse aspects of synaptic development and function at the *Drosophila* larval NMJ (Jepson et al., 2014) that are linked with the DGC. Therefore, Dyb’s role in the ear may be through organising and recruiting proteins at cell junctions between support cells for these physiological roles.

Given the location of Dystrophin protein in JO, an attractive model for DGC function in the fly is that it serves a role in the integrity of the hemolymph-neuron barrier that is formed between the cap, scolopale and ligament cells *via* multiple septate junctions (Todi et al., 2005; Banerjee et al., 2006). The cellular junctions are controlled by protein complexes that assemble around transmembrane proteins such as DE-cadherin, Crumbs, and Neurexin IV which partners with the DGC (Tepass et al., 2001). Their role is critical for isolating the sensory dendrites and probably maintains the K^+^-rich receptor lymph bathing their apical cilia (Roy et al., 2013; Li et al., 2018). Such a function may be reflected in the equivalent blood-nerve barrier in the mammalian inner ear. Mutation of mouse α-Dystrobrevin caused defects in the blood-brain barrier that is formed by tight junctions (equivalent to fly septate junctions) (Lien et al., 2012). In humans and mice, sites of Dystrobrevin and DGC in the inner ear include Reissner, Spindle and Root cells (Orvis et al., 2021), which are involved in K^+^ maintenance in the cochlear endolymph (the functional equivalent of fly receptor lymph). The observed inclusions in the mitochondria of *Dyb* ^EY01099^ neuronal cells suggest indirectly affecting the exchange of ions and endolymph surrounding the neuronal bodies may affect the mitochondria. Similar inclusions in other studies have been shown by Energy Dispersive X-Ray Analysis to include crystallised calcium and phosphate reservoirs (Wolf et al., 2017). One role of mitochondria is to contribute to shaping intraneuronal calcium signals, but an excess of calcium could lead to neuronal death (Contreras et al., 2010). This response has been observed in different context including degenerative disease as Alzheimer (Calvo-Rodriguez et al., 2020) or in the inner ear hair cells after noise exposure (Vicente-Torres and Schacht, 2006; Wang et al., 2018).

Channels involved in maintenance of inner ear endolymph include AQP4 and Kir4.1, which are co-localised with α-Dystroglycan in mice (Takumi et al., 1998; Gu et al., 2020) and adult humans (Lopez et al., 2007). In addition, these channels have also been linked with podocyte filtration (Vogtlander et al., 2005) and the integrity of the blood-brain barrier (Moukhles et al., 2000). Some studies and co-localisation assays suggest that α-Dystroglycan is a laminin receptor (Montanaro et al., 1998). Both laminin and the DGC play a role in recruiting Kir4.1 and AQP4 channels in astrocytic cells for potassium buffering and water homeostasis (Nagelhus et al., 1999; Guadagno and Moukhles, 2004). The co-expression of Kir4.1 and α-dystroglycan in the stria vascularis suggests a similar role in the inner ear (Orvis et al., 2021). This idea is also sustained in studies performed in Dystrophin knockout mice (*mdx*) that showed that cell junction molecules like connexin 43 show aberrant localisations and a disruption of ion gradients (Himelman et al., 2020; Valera et al., 2021). The suppression of connexin 43 leads to an increase in stria permeability (Zhang et al., 2020). In *Drosophila*, there are eight paralogs of the aquaporin family of genes, including *Drip* and *big brain* (*bib*). However, data as to location and function of these channels are scarce (Limmer et al., 2014).

In mouse neuronal/glial cells, α-Dystrobrevin and syntrophins recruit neuronal nitric oxide synthase (NOS) as well as other unidentified voltage-gated ion channels and kinases (Albrecht and Froehner, 2002). NOSs are crucial players for hearing loss, linked to different pathologies (Heinrich and Helling, 2012). Previous studies have shown that neuronal NOS (nNOS) is significantly reduced at the muscle membrane in α-dystrophin-deficient mice and an artificial increase of Nitric Oxide (NO) in these mice protects the muscle from degeneration (Wehling et al., 2001). In α-Dystrobrevin knockouts mice, the DGC is correctly localised, but the signalling protein NOS is displaced from the membrane, and signalling is impaired, specifically nNOS shows low levels (Grady et al., 1999). Interestingly, altered levels of inducible NOS (iNOS) and nNOS are upregulated in spiral ganglion neurons (Michel et al., 2000; Watanabe et al., 2001), matching with high levels of iNOS found in MD patients (Ishiyama et al., 2018). Additionally, in MD patient utricles, a downregulation of collagen IV and laminin-beta, both molecules linked with the DGC, was found (Calzada et al., 2011). These data suggest that the NOS pathway may be related to the hydrops and the spiral ganglion degeneration found in MD patients (Schuknecht, 1991) and the induced endolymphatic hydrops in animals (Bixenstine et al., 2008).

Both channel distribution and NOS could suggest that DGC has a role in endolymph homeostasis. In mice, endolymph volume is regulated by the stria vascularis and Reissner’s membrane. RNAseq data from mice stria vascularis confirmed that DGC components are present on several stria cells (Gu et al., 2020). The major DGC components, Dystroglycan, Dystrobrevin, and Dystrophin, present a diffuse expression pattern and only coincide in the root cells in the stria vascularis base. The root cell plays different roles within the normal homeostatic function, including cation absorption from the endolymph and K^+^ transport between junctional gap compartments (Jagger and Forge, 2013). In addition, Dystrobrevin is present in the marginal cells derived from the otic epithelium, whereas Dystrophin and Dystroglycan show higher expression in the intermediate cells derived from the migratory neural crest. Just like the supporting cells of JO maintaining K^+^ homeostasis of the scolopidial receptor lymph, the marginal, intermediate and basal cells of the mammalian cochlea are important for K^+^ homeostasis of the cochlear endolymph (Wangemann, 2002; Nin et al., 2008; Hibino et al., 2010; Trune, 2010).

### Implications for Meniere’s disease in humans

Despite new advances in aetiology, the pathophysiology of MD remains controversial. However, there are aspects of the disease that may be highly relevant to our findings. The dominant hypothesis is idiopathic endolymphatic hydrops (EH), defined as increased hydraulic pressure within the inner ear endolymphatic system and a possible break in the membrane that separates the perilymph from the endolymph (Teggi et al., 2019). The resultant chemical mixture bathes the receptors (Boudewyns and Claes, 2001), leading to a depolarisation blockade and transient loss of function. Although EH is the only consistently found anatomical abnormality in many MD patients, this correlation does not imply causality (Cureoglu et al., 2016). The reason is that EH has also been found in healthy individuals without MD symptoms (Merchant et al., 2005). Some investigators have therefore questioned whether EH is a marker rather than a cause. The relation between MD and hydrops is generally studied using induced endolymphatic hydrops animals (Salt and Plontke, 2010). These studies have suggested the link with NOS dysregulation. However, genetic studies in large MD patient cohorts showed that functional variants in NOS1 and NOS2A are not associated with progressive hearing loss in MD (Gazquez et al., 2011; Teranishi et al., 2013). Our results suggest that the link between the NOS pathway, hydrops and MD could be the DGC complex and alterations in the homeostatic stability of the system.

Our findings that Dyb and DGC are predominant in JO support cells required to maintain the receptor milieu is therefore highly significant. In *Drosophila*, we have observed changes in the distribution of the NompC channel at the pupal state. If there is a reduction in NompC at adult stages, this would be linked with the hearing loss observed. However, there is no reason to think that a reduction of NompC is essential to explain a mechanogating change. Although not directly equivalent, a similar alteration might be predicted for Kir4.1 and AQP4 in the mammalian inner ear. In addition, the mitochondrial inclusions may reflect neurons under physiological stress, reinforcing the hypothesis of alteration in the endolymph homeostasis. Alteration in channel distributions and endolymph homeostasis could explain the hydrops presence in human patients.

In the mammalian inner ear, both the auditory and the vestibular system use hair cells as their receptors but they have many differences that specializes them for appropriate coding of different sensory modalities (Sadeghi and Geleoc, 2022). In *Drosophila*, the JO comprises entirely separate subsets of chordotonal neurons controlling hearing and gravity + wind detection (Kamikouchi et al., 2009). Our results suggest that the gravity neurons could be affected first, albeit subtly, because they are only defective under dark conditions. Subsequently, the hearing neurons are affected, generating the hearing loss detected by electrophysiology. Interestingly, the course of the phenotype matches MD, where the first symptom detected in the clinic is the vestibular attack, with hearing loss occurring much later.

Another MD characteristic is that symptoms do not progress smoothly over time but show fluctuation. Our *Drosophila* data have shown a decay in some aspects of JO function as the adults age, but we were not able to observe whether fluctuations occur due to the need for averaging across flies. It is possible, however, that fluctuations reflect a sensitivity of DGC-associated cell to environmental stress. MD patients have, for example, reported repeated and intense vertigo crises after certain triggers (Soderman et al., 2004). The *Dyb* fly model has potential to provide a platform for exploring the effect of physiological stress on the fluctuation and progression of phenotypes.

Our results have shown that despite Dyb protein presenting only a 45%–52% identity of amino acid sequence with α-Dystrobrevin, differential effects of their mutation on hearing and balance appear to be preserved. Although the *Drosophila* model is a powerful tool to study the molecular mechanisms behind the MD candidate genes as *DTNA*, there are also limitations. The main limitation of our model is that *Drosophila* mutants are null or knockdown, so they take effect during development, and this can explain why we observe the MD-like phenotype from early ages, whereas in humans, MD usually appear later in life. Therefore, more studies would be needed using adult-specific knockdown to determine whether alteration in homeostasis appears after the developmental stage.

This question is of particular interest as our data show that auditory amplification (power gain) in *Dyb* ^EY01099^ flies is statistically indistinguishable controls at day 2 (although a tendency to lower levels might be observable). Later, at day 10 and day 20, the *Dyb* ^EY01099^ show a significantly lower power gain than controls, at day 30 however levels are similar to controls again. These results match with a progressive form of hearing loss where age-related loss of auditory amplification arrives sooner than normal, probably due to a homeostatic defect. The homeostatic mechanism - which buffers the system against a loss of summary functional output—is likely defective from day 1. The statistically identical power gain at day 2 (Figure 3) shows that the homeostatically defective mutants can still provide a ‘normal’ overall amplification at young age, but—as Figure 4 shows - they already have to struggle harder than their controls. Already at day 2, the number of sensitive auditory transducers is roughly half of the controls. This is compensated by higher single channel gating forces, and probably other system parameters, which we cannot assess right now. Overall, this homeostatic compensation leads to an almost normal functional output for some time. Some system functions, however, may not be fully restored (e.g. those required in the climbing assay).

Another limitation of our study is the small endolymph space of the *Drosophila* JO, which impedes the analysis of filtration aspects. However, it is conceivable that mouse models will provide further data on ion channel distribution and endolymph distribution. The last limitation is that the amino acids affected by *DTNA* mutations described to date are not conserved in *Drosophila,* so their pathogenicity cannot be tested directly. However, future genetic studies in large cohorts may reveal variants or genes that could be tested in *Drosophila*.

### Implications for dystrophin-glycoprotein complex role more widely

Dystrophin is well known to be mutated in Duchenne and Becker muscular dystrophies. Apart from muscular phenotypes, it is responsible for CNS effects such as mild cognitive impairment. Hearing loss has not been well researched, but has been demonstrated in various forms of muscular dystrophy (Mburu et al., 2003; Morioka et al., 2020) The *mdx* mouse does not show hearing loss (Pillers et al., 1999), but exhibits enhanced vulnerability to acoustic trauma (Chen et al., 2002). Confirming the link between DGC, hearing loss and physiological stress may help inform molecular pathophysiology and clinical dysfunction in muscular dystrophies as well as MD.

## Data Availability

The raw data supporting the conclusion of this article will be made available by the authors, without undue reservation.
