# Supplementary material for: A Drosophila model for Meniere’s disease: Dystrobrevin is required for support cell function in hearing and proprioception
Source: Front Cell Dev Biol. 2022 Nov 10;10:1015651. doi: 10.3389/fcell.2022.1015651 (PMC9688402; doi:10.3389/fcell.2022.1015651)
Supplement: Supplementary file 1 [file DataSheet1.pdf]

Supplementary Material

1 Supplementary Figures and Tables

1.1 Supplementary Figures

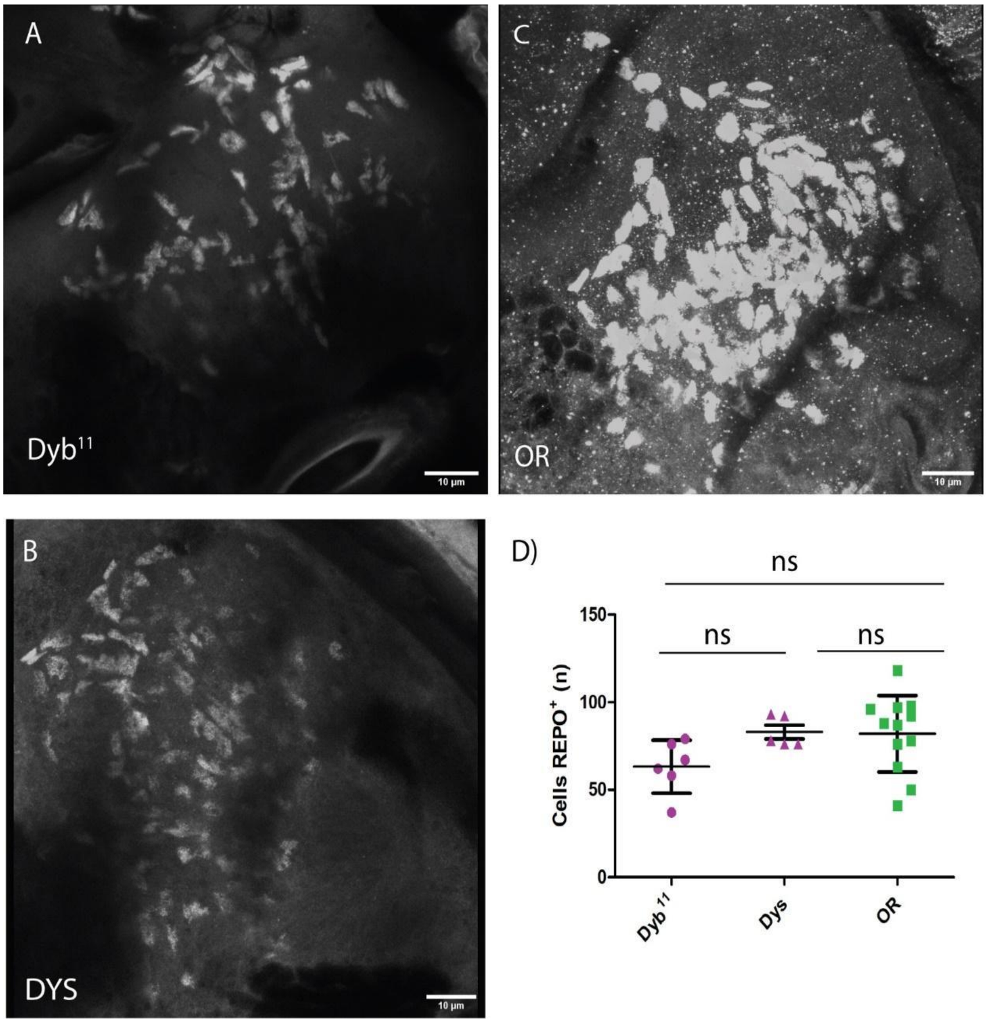

Supplementary Figure 1. REPO-positive cells in confocal images of immunofluorescence in pupal antennae.

Immunostaining with anti-REPO antibodies. **A)** *Dyb^{11}* mutant (n=6) **B)** *Dys* mutant (n=5) **C)** Immunostaining in the control OR (n=12). **D)** Graph of total REPO positive cells in each JO z-stack; each dot represents a JO, horizontal line represents the mean and vertical line SD. Data passed the normality test, and so were compared using a 1-way ANOVA and Tukey's Multiple Comparison Test. The REPO cell count showed a trend to reduction in the *Dyb^{11}* compared with the control and *Dys*, but no significant differences were found.

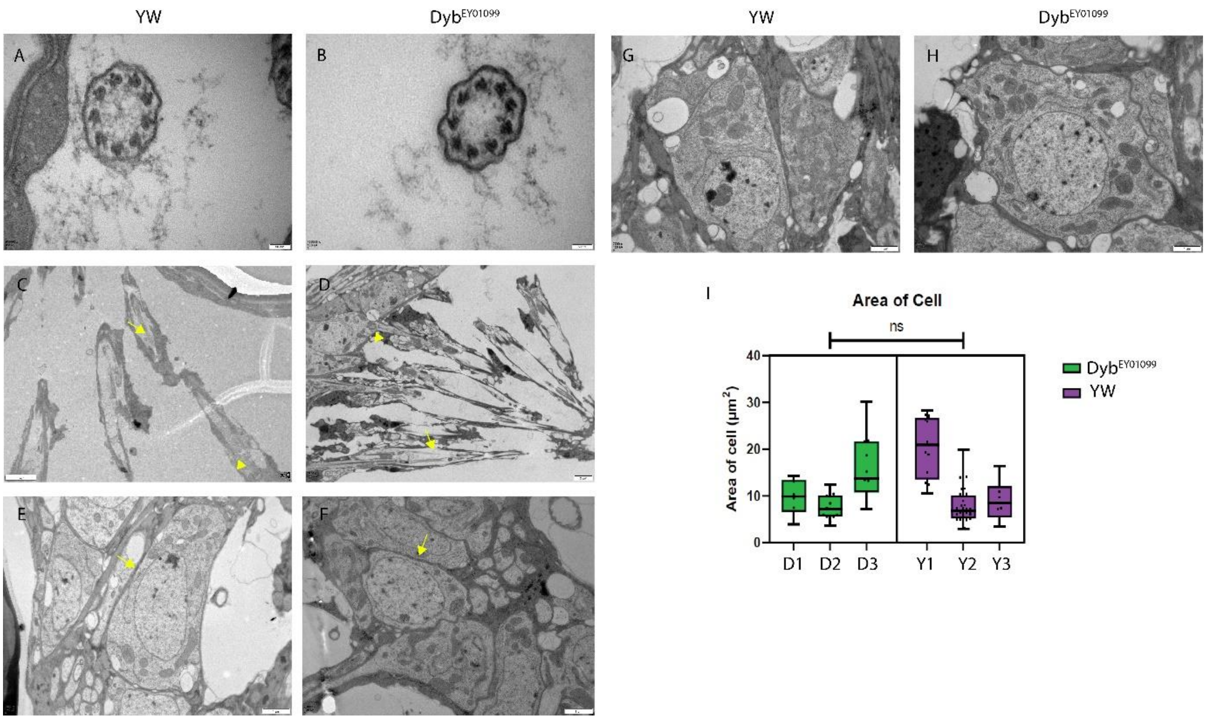

Supplementary Figure 2. $Dyb^{EY01099}$ does not present ultrastructural differences from *yw* control

A-B) Transmission electron micrographs of antennae from $Dyb^{EY01099}$ (A) and *yw* (B) adults, neuronal cilia transverse sections. In both $Dyb^{EY01099}$ and *yw* the ciliary microtubule doublets are intact, showing a 9+0 arrangement, with attached outer and inner dynein arms. **C-D)** Longitudinal sections through *yw* (C) and $Dyb^{EY01099}$ (D) scolopidia. Within the scolopales are complete ciliary structures with the distal basal bodies (arrowhead) and ciliary dilations (arrow). The scolopale structures appear similar. **E-F)** Neuronal cell body transverse sections of *yw* (E) and $Dyb^{EY01099}$ (F), showing that the septate junction is present in both genotypes at an early age. **G-H)** Neuronal cell body transverse sections of *yw* (G) and $Dyb^{EY01099}$ (H), showing little difference in overall appearance. **I)** Comparison of the area of the neuronal cell sections. The number of sections analysed of individual heads was Y1=7, Y2=14 Y3=11 for *yw* and D1=15, D2=36 D3=6 for $Dyb^{EY01099}$. Boxplots with the median values, points values of individual heads (*yw* (Y1-3) and $Dyb^{EY01099}$ (D1-3)) and quartile values as horizontal lines. The two conditions have similar spreads of data with means of 11.06 µm2 and 12.40 µm2 in YW and $Dyb^{EY01099}$ conditions, respectively. No difference between the groups is supported by a nested t-test (Nested t-test: $t_4 = 0.2822$, $F_{1,4} = 0.07966$, $p= 0.7918$). (NS: meaning $P > 0.05$).

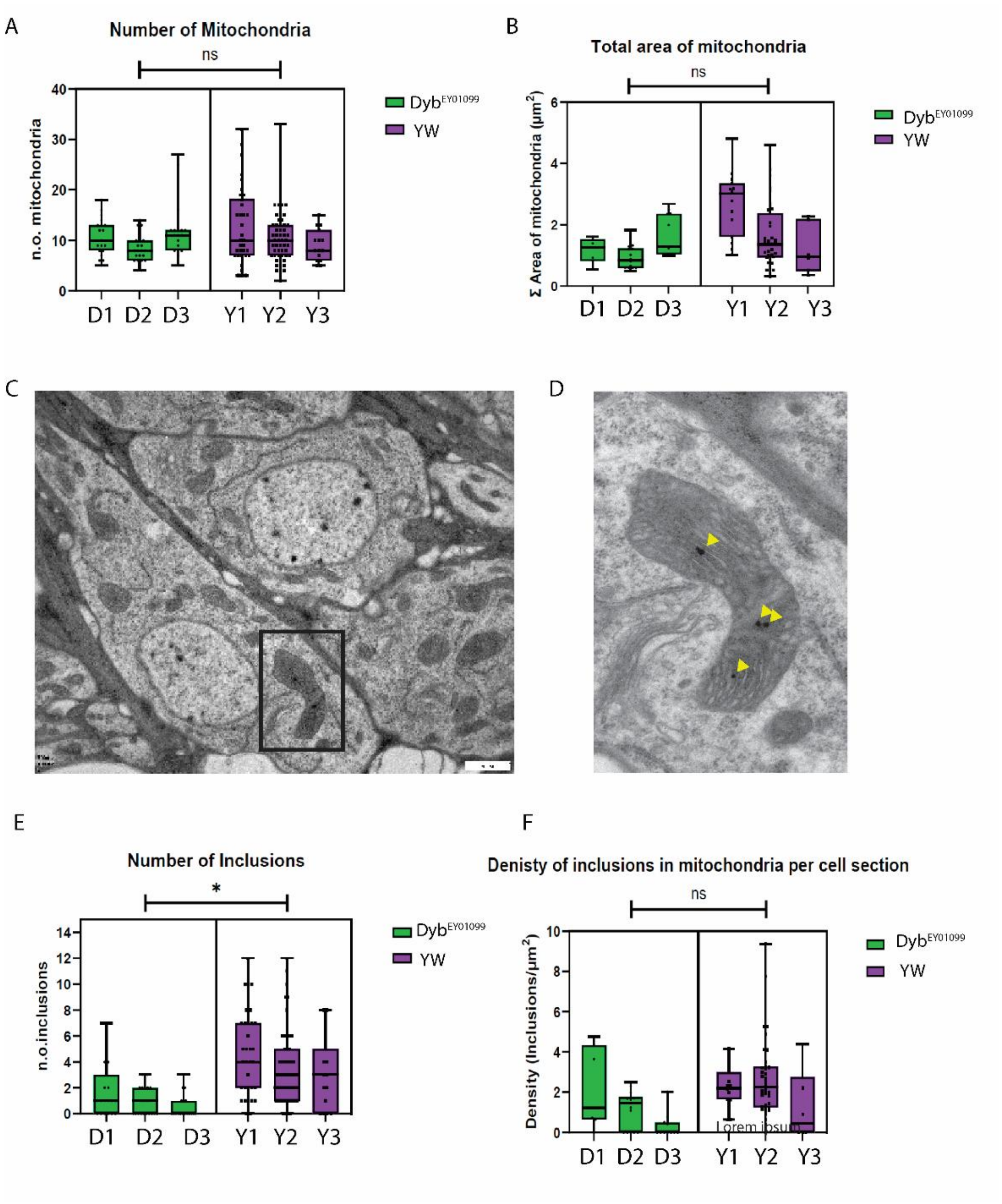

Supplementary Figure 3. Mitochondrial comparison between *Dyb*^{EY01099} and *yw*

Transmission electron micrographs of antennae from *Dyb*^{EY01099} and *yw* adults. **A)** Number of mitochondrial sections in neuronal cell cross-sections. A nested t-test showed no-significant difference in the median values between *Dyb*^{EY01099} and *yw* (Nested t-test: t4=0.5660, F1,4=0.3204, p=0.6016). **B)** Total area of mitochondrial sections in neuronal cell cross-sections. Overall, total area *Dyb*^{EY01099} is slightly larger (median values =2.690, 1.621, 1.207 μm^2), compared to the *yw* control (median values =1.155, 0.9303, 1.703 μm^2). This is only a trend as a nested t-test reveals the difference is non-significant (Nested t-test: t4=1.229, F1,4=1.510, p=0.2864). **C-D)** TEM images showing dense particle inclusions in *Dyb*^{EY01099} neuronal mitochondria**. D)** Zoom of the area marked with the black square in (C). Inclusions are indicated with arrowheads. **E)** Plot of number of inclusions. *Dyb*^{EY01099} neuronal mitochondria have a larger number of inclusions (median values=4.636, 3.473, 3.133), compared to the *yw* control (median values = 1.789, 1.045, 0.5556). A nested t-test revealed a significant difference between the two groups (Nested

t-test: t4=4.569, F1,4=20.88, p=0.0103). **F)** Density of inclusions in mitochondria per cell section. Overall, the *Dyb*^{EY01099} has a trend towards a greater density of inclusions (median values = 2.196, 2.695 and 1.254 inclusions/µm^2), compared to the *yw* control (median values=2.192, 1.134 and 0.3387 inclusions/µm^2), but a nested t-test revealed no significant difference among the groups (Nested t-test: t4=1.646, F1,4=2.708, p=0.1752). All the boxplots and values from individual cell sections (points) depicting the number of mitochondria within two conditions (*yw* control antennae (Y1-3) and *Dyb*^{EY01099} mutant antenna (D1-3)), n=3. The boxplot indicates the median and quartile values as horizontal lines. (NS : meaning P > 0.05 and * means P ≤ 0.05). For B and F the number of sections analysed of individual heads was Y1=7, Y2=14 Y3=11 for *yw* and D1=15, D2=36 D3=6 for *Dyb*^{EY01099}. For A and B the number of sections analysed of individual heads was Y1=19, Y2=22 Y3=18 for *yw* and D1=33, D2=55 D3=15 for *Dyb*^{EY01099}.

1.2 Supplementary Tables

Table S1: Primer used to generate the GFP-enhancer transgenic reporter line

	Target	Primers	Fragment Size	Notes
**First primer set (first fragment):**	First Dyb FW	GGTACCgtccggattatattaccaaa	3401	EXTRA + KpnI+Left
	First Dyb RV	TCTAGAtgggcagtctagttggcttt		EXTRA + XbaI+Right
**Second primer set (second fragment):**	Second Dyb FW	GGTACCactgtctgcagctggacatg	3348	EXTRA + KpnI+Left
	Second Dyb RV	TCTAGAgggaaatcaaagccaaacaa		EXTRA + XbaI+Right
**Third primer set (third fragment):**	Third Dyb FW	GGTACCacggaataaatccgggtctc	3433	EXTRA + KpnI+Left
	Third RF	TCTAGAgcattatgatcaggataagcgtaa		EXTRA + XbaI+Right

Table S2: Dunn's Multiple Comparison. Test under light conditions.
Significance on table is signified by asterisks: *, P ≤ 0.05; **, P ≤ 0.01; ***, P ≤ 0.001

Dunn's Multiple Comparison Test Light	Significant? P < 0.05?	Significance
Oregon-R vs Fd3F	Yes	***
Oregon-R vs YW	No	ns
Oregon-R vs $Dyb^{EY01099}$	No	ns
Oregon-R vs W^{1118}	No	ns
Oregon-R vs Dyb^{11}	Yes	*
Oregon-R vs Dys	No	ns
Fd3F vs YW	Yes	***
Fd3F vs $Dyb^{EY01099}$	Yes	***
Fd3F vs W^{1118}	Yes	*
Fd3F vs Dyb^{11}	No	ns
Fd3F vs Dys	No	ns
YW vs $Dyb^{EY01099}$	No	ns
YW vs W^{1118}	No	ns
YW vs Dyb^{11}	Yes	*
YW vs Dys	No	ns
$Dyb^{EY01099}$ vs W^{1118}	No	ns
$Dyb^{EY01099}$ vs Dyb^{11}	Yes	***
$Dyb^{EY01099}$ vs Dys	Yes	**
W^{1118} vs Dyb^{11}	No	ns
W^{1118} vs Dys	No	ns
Dyb^{11} vs Dys	No	ns

frontiers

Table S3: Dunn's Multiple Comparison. Test under Dark conditions.
Significance on table is signified by asterisks: *, P ≤ 0.05; **, P ≤ 0.01; ***, P ≤ 0.001

Dunn's Multiple Comparison Test Dark	Significant? P < 0.05?	Significance
Oregon-R vs Fd3F	Yes	***
Oregon-R vs YW	No	ns
Oregon-R vs $Dyb^{EY01099}$	No	ns
Oregon-R vs W^{1118}	No	ns
Oregon-R vs Dyb^{11}	No	ns
Oregon-R vs Dys	Yes	*
Fd3F vs YW	Yes	***
Fd3F vs $Dyb^{EY01099}$	No	ns
Fd3F vs W^{1118}	Yes	***
Fd3F vs Dyb^{11}	No	ns
Fd3F vs Dys	No	ns
YW vs $Dyb^{EY01099}$	Yes	*
YW vs W^{1118}	No	ns
YW vs Dyb^{11}	Yes	***
YW vs Dys	Yes	***
$Dyb^{EY01099}$ vs W^{1118}	No	ns
$Dyb^{EY01099}$ vs Dyb^{11}	No	ns
$Dyb^{EY01099}$ vs Dys	No	ns
W^{1118} vs Dyb^{11}	Yes	*
W^{1118} vs Dys	Yes	**
Dyb^{11} vs Dys	No	ns
